# Loss-of-function variants in *KCTD19* cause non-obstructive azoospermia in humans

**DOI:** 10.1016/j.isci.2023.107193

**Published:** 2023-06-28

**Authors:** Junyan Liu, Fazal Rahim, Jianteng Zhou, Suixing Fan, Hanwei Jiang, Changping Yu, Jing Chen, Jianze Xu, Gang Yang, Wasim Shah, Muhammad Zubair, Asad Khan, Yang Li, Basit Shah, Daren Zhao, Furhan Iqbal, Xiaohua Jiang, Tonghang Guo, Peng Xu, Bo Xu, Limin Wu, Hui Ma, Yuanwei Zhang, Huan Zhang, Qinghua Shi

**Affiliations:** 1 Division of Reproduction and Genetics, First Affiliated Hospital of USTC, Hefei National Research Center for Physical Sciences at the Microscale, the CAS Key Laboratory of Innate Immunity and Chronic Disease, School of Basic Medical Sciences, Division of Life Sciences and Medicine, Biomedical Sciences and Health Laboratory of Anhui Province, Institute of Health and Medicine, Hefei Comprehensive National Science Center, University of Science and Technology of China, Hefei 230027, China; 2 Institute of Pure and Applied Biology, Zoology Division, Bahauddin Zakariya University, Multan 60800, Pakistan; 3 Hainan Jinghua Hejing Hospital for Reproductive Medicine, Hainan 570125, China

**Keywords:** health sciences, biological sciences, Biochemistry

## Abstract

Azoospermia is a significant cause of male infertility, with non-obstructive azoospermia (NOA) being the most severe type of spermatogenic failure. NOA is mostly caused by congenital factors, but our understanding of its genetic causes is very limited. Here, we identified a frameshift variant (c.201_202insAC, p.Tyr68Thrfs∗17) and two nonsense variants (c.1897C>T, p.Gln633∗; c.2005C>T, p.Gln669∗) in *KCTD19* (potassium channel tetramerization domain containing 19) from two unrelated infertile Chinese men and a consanguineous Pakistani family with three infertile brothers. Testicular histological analyses revealed meiotic metaphase I (MMI) arrest in the affected individuals. Mice modeling *KCTD19* variants recapitulated the same MMI arrest phenotype due to severe disrupted individualization of MMI chromosomes. Further analysis showed a complete loss of KCTD19 protein in both *Kctd19* mutant mouse testes and affected individual testes. Collectively, our findings demonstrate the pathogenicity of the identified *KCTD19* variants and highlight an essential role of *KCTD19* in MMI chromosome individualization.

## Highlights

• *KCTD19* pathogenic variants were found in infertile men with meiotic metaphase I arrest
• *Kctd19* mutant mice recapitulate meiotic defects observed in affected individuals
• KCTD19 deficiency disrupts meiotic metaphase I chromosomes individualization in mice

## Graphical abstract

**Figure:**
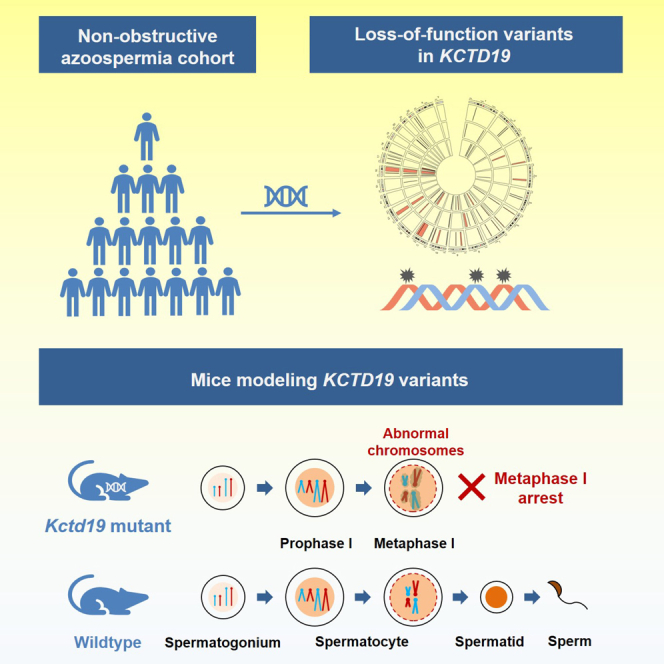

## Introduction

Infertility has become a global reproductive health problem, affecting approximately 10% of couples of childbearing ages, with male factors accounting for approximately 50% of them. Azoospermic individuals represent approximately 15% of all infertile male cases worldwide, with non-obstructive azoospermia (NOA) representing approximately 60% of azoospermic cases.^1^ However, the pathogenesis in approximately 85% of men with NOA is unknown, and the genetic and molecular basis underlying NOA remains to be elucidated.^2^ Thousands of genes have been implicated in spermatogenesis, with more than 400 genes specifically linked to male infertility in knockout mouse models.^3^ However, due to the high heterogeneity of spermatogenic failure found in NOA-affected individuals, only a few genetic pathogenic variants have been reported to be the genetic causes for NOA-affected individuals.^2^

The family of potassium (K+) channel tetramerization domain (KCTD) proteins has 26 members (KCTD1–21, KCTD12B, TNFAIP1, KCNRG, SHKBP1, and BTBD10) in humans and is characterized by the N-terminal BTB/POZ domain.^4^ KCTD proteins have been implicated in a variety of biological processes, including transcriptional repression,^5^^,^^6^ gating of the voltage-gated potassium channel,^7^ and interaction with the cullin E3 ubiquitin ligase complex.^8^^,^^9^ Pathogenic variants in *KCTD* genes have been reported in various human diseases,^10^ such as scalp-ear-nipple syndrome (*KCTD1*),^11^ neurocognitive disorders (*KCTD3*),^12^ neurodevelopmental disease (*KCTD7*),^13^^,^^14^ bipolar disorder (*KCTD12*), autism and schizophrenia (*KCTD13*), movement disorder (*KCTD17*), and obesity (*KCTD15*).^15^ In addition, most of the KCTD family proteins have also been associated with the occurrence and progression of different types of cancers, such as leukemia, liver cancer, and breast cancer.^16^

Among KCTD members, KCTD19 is the only protein showing testis-enriched expression and was first identified in a germ cell-specific complex with ZFP541 and HDAC1. This complex was speculated to function in chromatin remodeling during spermiogenesis by histone deacetylation.^17^ KCTD19 was reported to be essential for meiotic prophase completion during mouse spermatogenesis by forming a meiosis-specific transcriptional repressor complex with ZFP541, DNTTIP1, and HDAC1/2 to regulate gene expression.^18^ Male *Kctd19* knockout mice are infertile due to meiotic metaphase I (MMI) arrest.^18^^,^^19^^,^^20^ Recently, *KCTD19* variants were identified in different NOA cohorts,^21^ however, the specific variants’ information and whether these variants cause human spermatogenic failure have not been explained in detail. The relationship between *KCTD19* pathogenic variants and human fertility remains uncertain.

In the present study, we performed whole-exome sequencing (WES) in Chinese sporadic NOA-affected men with unexplained meiotic arrest and identified two homozygous loss-of-function variants in *KCTD19* (GenBank: NM_001100915). Next, we screened infertile men born in consanguineous families from the Pakistani population and identified homozygous nonsense variants in *KCTD19* that recessively co-segregated with infertility within the family. Histological analyses of testes of the affected men showed spermatocyte development arrested at MMI with no postmeiotic germ cells detected. Two *Kctd19* mutant mouse lines mimicking these variants recapitulated the meiotic defects of NOA-affected men, due to the loss of KCTD19 protein. Furthermore, severe disrupted individualization of MMI chromosomes was observed in *Kctd19* mutant mice. These findings collectively demonstrate a causal relationship between loss-of-function variants in *KCTD19* and autosomal recessive NOA and male infertility.

## Results

### Identification of *KCTD19* variants in NOA-affected individuals

This study focuses on two sporadic azoospermia-affected individuals (P7864 and P2034), as well as a consanguineous family (Family-01) with three infertile brothers. All the affected individuals had normal semen volumes, but no sperm were found in their ejaculates. No sign of obstructive forms of azoospermia or absence of vas deference was found by palpation. Thus, azoospermia is more likely to be caused by spermatogenic failure than obstruction in these individuals, and the diagnosis of NOA was suggested.^22^ Additionally, they all showed normal serum levels of testosterone, follicle-stimulating hormone (FSH), and luteinizing hormone (LH). Both P7864 and P2034 had normal prolactin levels, while prolactin levels were not tested in affected individuals from Family-01. The clinical information and test results for each affected individual are summarized in Table 1. They have a normal karyotype (46,XY) and no microdeletion of the Y chromosome. Thus, we performed WES on DNA samples from P7864, P2034, and Family-01 members to investigate the genetic causes of their infertility.

**Table 1:** Clinical characteristics of the cases

	Reference values	P7864	P2034	Family-01		
				IV:1	IV:3	IV:5
Age (years)^a^	–	31	34	29	26	18
Height/weight (cm/kg)	–	167/78	164/80	168/70	168/85	168/87
Karyotype	–	46,XY	46,XY	46,XY	46,XY	46,XY
Diagnosis of disease	–	NOA^d^	NOA	NOA	NOA	NOA

**Semen analysis**^b^						

Semen volume (mL)	>1.5	3.0	2.0	4.0	2.0	3.8 ± 0.8
Sperm concentration (millions/mL)	>15	0	0	0	0	0

**Hormone analysis**^c^						

Testosterone (ng/dL)	249.0–836.0	286.8	280.6	–	–	–
FSH (mIU/mL)	1.4–15.4	12.4	13.6	9.2	12.5	13.4
LH (mIU/mL)	1.2–7.8	5.8	3.6	3.2	5.0	5.7
Prolactin (ng/mL)	3.0–14.7	8.6	7.5	–	–	–
Testis size (mL)	>12.5	14.0	10.0	–	–	–

FSH, follicle-stimulating hormone; LH, luteinizing hormone.

a Ages at diagnosis.

b Reference values were published by WHO in 2010.

c Reference values were suggested by the local clinical laboratory.

d NOA, Non-obstructive azoospermia.

**Figure 1:**
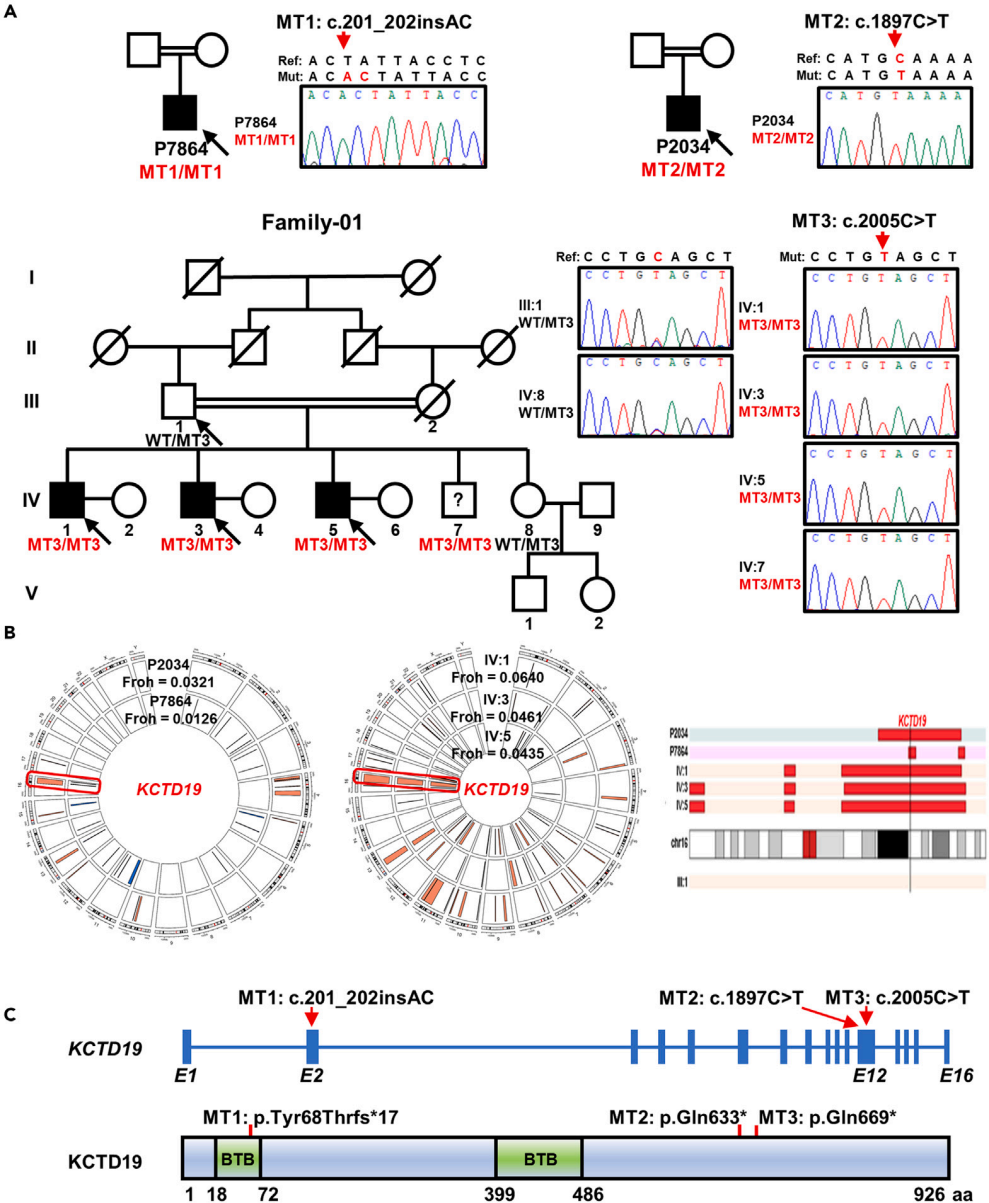
Identification of homozygous variants in *KCTD19* from NOA affected individuals (A) The pedigree charts of the families. Double horizontal lines represent the consanguineous unions. Squares and circles denote male and female members, respectively. Solid symbols indicate affected members, and open symbols denote unaffected members. Slashes represent deceased members. Members indicated by black arrows were selected for whole-exome sequencing. Sanger sequencing chromatograms of *KCTD19* are shown on the right side. Red arrows indicate the corresponding variants. MT, variant. WT, wild-type. ?, individual with unknown reproductive status. (B) Runs of homozygosity (ROH) analysis of affected individuals. Left and middle panels show the distribution of homozygous regions in the genome of P2034 and P7864. Right panel shows ROH on chromosome 16. The histograms highlighted in red represent the estimated ROH. The black vertical bar indicates the *KCTD19* locus. (C) Genomic and protein structure of KCTD19, showing the variant positions with red arrows or red bars. The schematic of the gene composition is based on the GenBank database (GRCh37, transcript ID: NM_001100915) and blue solid squares represent exons (E). The schematic of the protein composition is based on the UniProt database (Q562E2) and green solid squares represent BTB (broad complex, tram-track and bric-a-brack) domains.

Three potentially pathogenic variants in *KCTD19* were identified (Figures 1A and S1). P7864 carried a homozygous frameshift variant (MT1, chr16 [GRCh37]: g.67354590A>AGT; NM_001100915:c.201_202insAC, p.Tyr68Thrfs∗17). This variant maps to a BTB domain common among KCTD proteins. P2034 carried a homozygous nonsense variant (MT2, chr16[GRCh37]: g.67327768G>A; NM_001100915: c.1897C>T, p.Gln633∗). Both *KCTD19* variants were validated at the genomic DNA and cDNA levels by Sanger sequencing (Figures 1A and S2). For consanguineous Family-01, we performed WES on three infertile brothers (Ⅳ:1, Ⅳ:3, and Ⅳ:5) and their father. A homozygous *KCTD19* nonsense variant (MT3, chr16[GRCh37]: g.67327660G>A; NM_001100915: c.2005C>T, p.Gln669∗) was identified (Figure 1A). Sanger sequencing analysis using genomic DNA and cDNA confirmed that MT3 was homozygous in affected individuals and heterozygous in their father, thus cosegregating with male infertility in the family (Figures 1A and S2). Notably, the 17-year-old brother Ⅳ:7 was also homozygous for the *KCTD19* variants, but he was unwilling to provide semen samples or participate in any further clinical examinations because he was young.

All NOA-affected individuals involved in our study were offspring of consanguineous marriages and autozygosity mapping confirmed that all three *KCTD19* variants were located within the homozygosity regions (Figure 1B), which supports their pathogenicity. Meanwhile, all three *KCTD19* variants were absent in the general population databases (1000 Genomes Project, ESP6500, and gnomAD), thus providing further genetic evidence of the pathogenicity of these variants (Table S1). Furthermore, both MT2 and MT3 were predicted to be highly pathogenic by Combined Annotation-Dependent Depletion (CADD) software, with Phred-scaled scores of 38 and 36, respectively, which means that both variants belong to the top 0.1% of the most pathogenic variants in the human genome.^23^ Based on the previous findings, all three variants were classified as “likely pathogenic” (MT1 and MT2) or “pathogenic” (MT3) according to the American College of Medical Genetics and Genomics (ACMG) guidelines (Table S2). Considering the available evidence presented previously and the infertile phenotype of *Kctd19* knockout mice,^18^^,^^19^^,^^20^ we propose that these *KCTD19* variants are the most likely pathogenic variants for male infertility in these individuals.

### Affected individuals displayed meiotic metaphase I arrest

**Figure 2:**
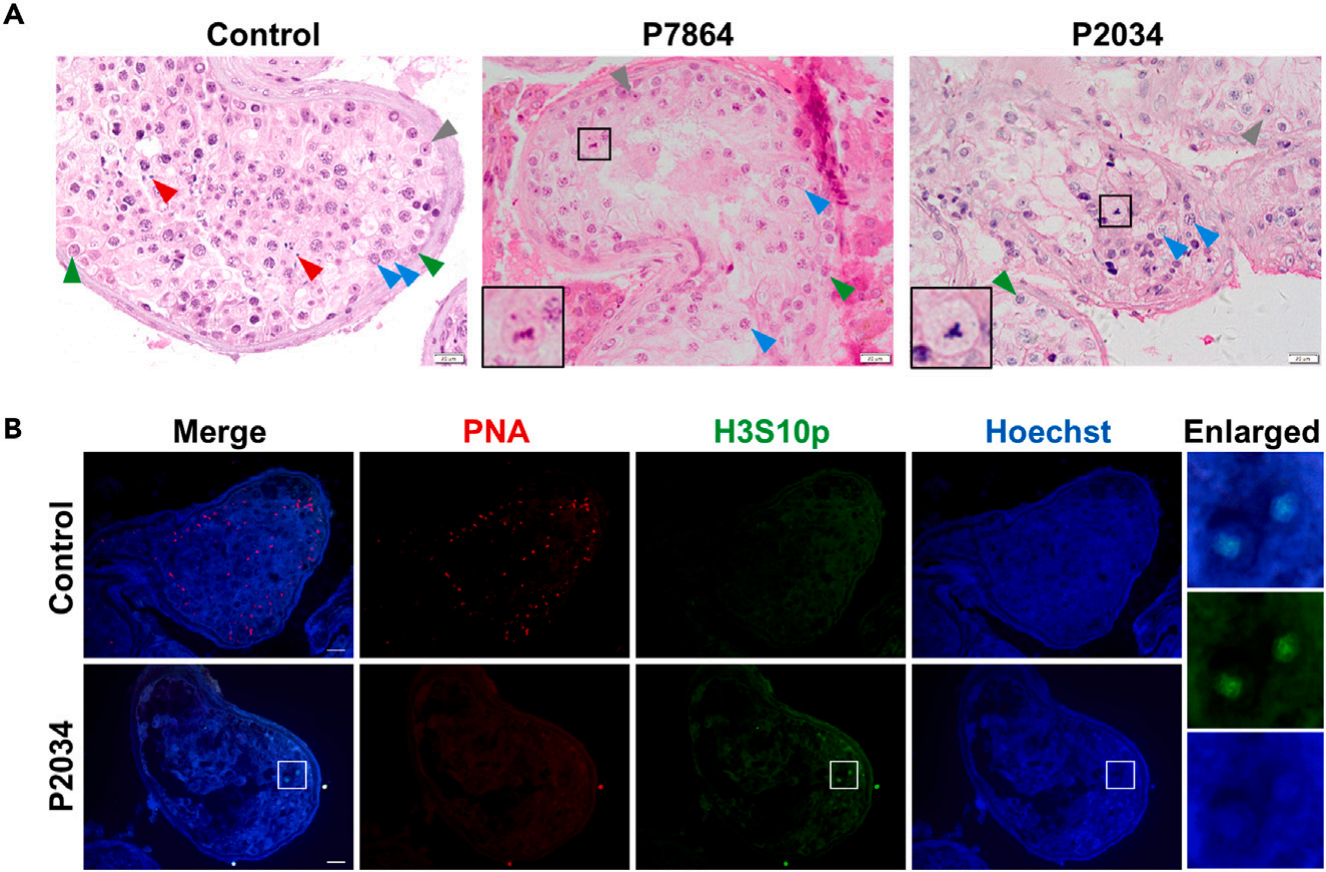
Individuals carrying *KCTD19* variants display meiotic metaphase I arrest (A) Histological analyses of testicular tissues by H&E staining. Black boxes are magnified views of metaphase cells. Green arrowheads indicate spermatogonia, blue arrowheads indicate spermatocytes, red arrowheads indicate spermatids, gray arrowheads indicate Sertoli cells. Scale bars indicate 20 μm. (B) Immunofluorescence staining of testicular sections from the control and P2034 with PNA (red) and an antibody against H3S10p (green), a marker for nuclei at division. The nuclei were stained with Hoechst 33342 (blue). The insets show magnified images of the field in white rectangles. Scale bars indicate 20 μm.

To examine the detailed meiotic defects of affected individuals carrying homozygous *KCTD19* variants, we performed hematoxylin and eosin (H&E) staining on testicular sections from P7864, P2034 and a man diagnosed with obstructive azoospermia (OA), serving as the control. Spermatogenic cells at all stages were observed in the seminiferous tubules of the control man, whereas in affected individuals, only spermatogonia and spermatocytes were found in all analyzed seminiferous tubules. Notably, the latest stage of spermatogenic cells that could be identified was MMI in both affected individuals, with no postmeiotic cells detected (Figure 2A). To confirm this, we then performed immunofluorescence staining with PNA, a marker of acrosomes in spermatids and sperm, and an antibody against phospho-Histone H3 at Ser-10 (H3S10p), a marker of metaphase chromosomes,^24^^,^^25^ on testicular sections from the control and P2034. In control testes, H3S10p-positive metaphase cells were rarely observed, while many PNA-positive spermatids were detected (Figure 2B). In contrast, no PNA signals were detected, while several H3S10p-positive cells lying adjacent to the lumen were found in testicular sections from P2034, indicating that spermatogenesis was arrested at the spermatocyte stage, with the most advanced germ cell types being metaphase cells (Figure 2B). These results indicated that affected individuals carrying *KCTD19* variants suffered from NOA because their spermatocytes failed to complete meiotic division.

### Effects of the identified variants on *KCTD19* expression

Considering that all these identified *KCTD19* variants introduce a premature stop codon (Figure 1C), which could result in mRNA degradation or truncated proteins, we thus performed reverse-transcription polymerase chain reaction (RT-PCR) to detect whether mutant *KCTD19* mRNA was present in our cases first. Although *KCTD19* mRNA levels are extremely low in tissues other than testis, they could still be detected by nested PCR in blood samples from the control and individuals (P7864, P2034, Ⅲ:1 and Ⅳ:1) carrying the heterozygous or homozygous *KCTD19* variants (Figure S2A). Sanger sequencing further validated these three variants at the mRNA level in the affected individuals (Figure S2B). To clarify whether these *KCTD19* variants result in truncated proteins, we transfected pEGFP-N1 vectors fused with wild-type (WT) or mutated KCTD19 into HEK293T cells and detected the fusion proteins by western blotting. The bands of fusion proteins were detected at their predicted molecular weights (Figure S3), indicating the existence of mutant KCTD19 proteins when exogenously expressed in cultured cells.

### *Kctd19* mutant mice recapitulated the meiotic metaphase I arrest phenotype of affected individuals

Based on the sequence alignment of the *KCTD19* coding and protein sequences between humans and mice, the identity was found to be 87% and 89%, respectively (Figure S4). All three identified *KCTD19* variants are conserved in the coding sequence between humans and mice (Figure S4). Thus, to further determine how these *KCTD19* variants impair spermatogenesis and male fertility *in vivo*, we generated two *Kctd19* mutant mouse lines according to the variants identified in the affected individuals, which were termed *Kctd19* ^*m1/m1*^ and *Kctd19* ^*m2/m2*^ mice, respectively (Figure S5A). *Kctd19*^*m1/m1*^ mice harbored a 20-bp deletion in exon 2, which led to a predicted truncated protein of 80 amino acids (p.Asp59Hisfs∗23), and mimics MT1 (p.Tyr68Thrfs∗17) from P7864 (Figure S5A). *Kctd19*^*m2/m2*^ mice possessed a 1-bp insertion in exon 12, which led to a predicted truncated protein of 648 amino acids (p.Thr631Asnfs∗19) and imitates MT2 and MT3 (p.Gln633∗ and p.Gln669∗) from P2034 and Family-01, respectively (Figure S5A). The *Kctd19* mutations in the two mutant mouse models were confirmed at the genomic and mRNA levels by Sanger sequencing (Figure S5).

**Figure 3:**
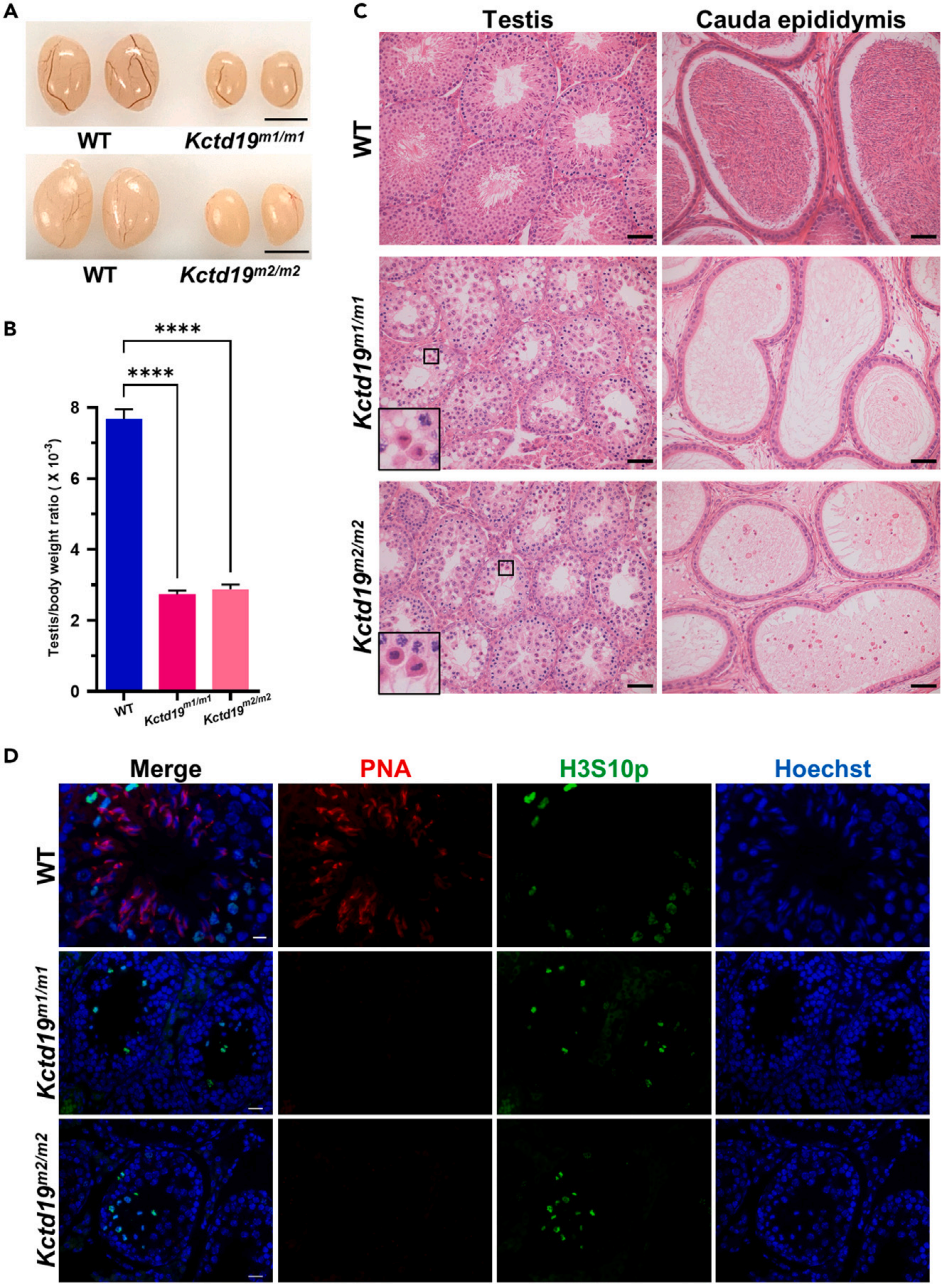
*Kctd19* mutant mice recapitulate the NOA phenotype of individuals homozygous for the *KCTD19* variants (A) Testis of 2-month-old WT, *Kctd19*^*m1/m1*^ and *Kctd19*^*m2/m2*^ mice. Scale bar indicates 4 mm. (B) Testis to body weight ratio of 2-month-old WT, *Kctd19*^*m1/m1*^ and *Kctd19*^*m2/m2*^ mice. Data are obtained from three or four mice for each genotype. The significance was determined via one-way ANOVA followed by Dunnett’s test. ∗∗∗∗p < 0.0001. (C) H&E staining of testicular and epididymal sections from adult WT, *Kctd19*^*m1/m1*^ and *Kctd19*^*m2/m2*^ mice. Black boxes indicate metaphase cells. Scale bars indicate 50 μm. (D) Immunofluorescence staining of testicular sections from adult WT, *Kctd19*^*m1/m1*^ and *Kctd19*^*m2/m2*^ mice with PNA (red) and an antibody against H3S10p (green). The nuclei were stained with Hoechst 33342 (blue). Scale bars indicate 20 μm.

*Kctd19*^*m1/m1*^ and *Kctd19* ^*m2/m2*^ mice showed normal growth and development. Two-month-old *Kctd19* ^*m1/m1*^ and *Kctd19* ^*m2/m2*^ males displayed reduced testis size and significant decrease in the testis to body weight ratio compared to their WT littermates (Figures 3A and 3B). Histological analyses revealed that seminiferous tubules of both *Kctd19* ^*m1/m1*^ and *Kctd19* ^*m2/m2*^ mice contained spermatogonia and primary spermatocytes but lacked postmeiotic germ cells, and consistently, no spermatozoa were observed in the cauda epididymides of either mutant mouse model (Figure 3C). As we previously performed for infertile men, testicular sections were stained for H3S10p and PNA to evaluate spermatogenesis in the mutant mice. In WT males, H3S10p-positive MMI spermatocytes and PNA-positive elongating spermatids were observed in stage Ⅻ seminiferous tubules (Figure 3D). However, accumulated MMI spermatocytes, but no PNA-positive germ cells, were found in the seminiferous tubules of *Kctd19* ^*m1/m1*^ and *Kctd19* ^*m2/m2*^ mice (Figure 3D), which was consistent with the phenotype of *Kctd19* knockout mice recently reported.^18^^,^^19^^,^^20^ These results suggested that the spermatogenesis of *Kctd19* ^*m1/m1*^ and *Kctd19* ^*m2/m2*^ mice was arrested at MMI, which recapitulated the MMI arrest phenotype of our affected individuals.

### *Kctd19* mutant mice showed abnormal MMI chromosome individualization

**Figure 4:**
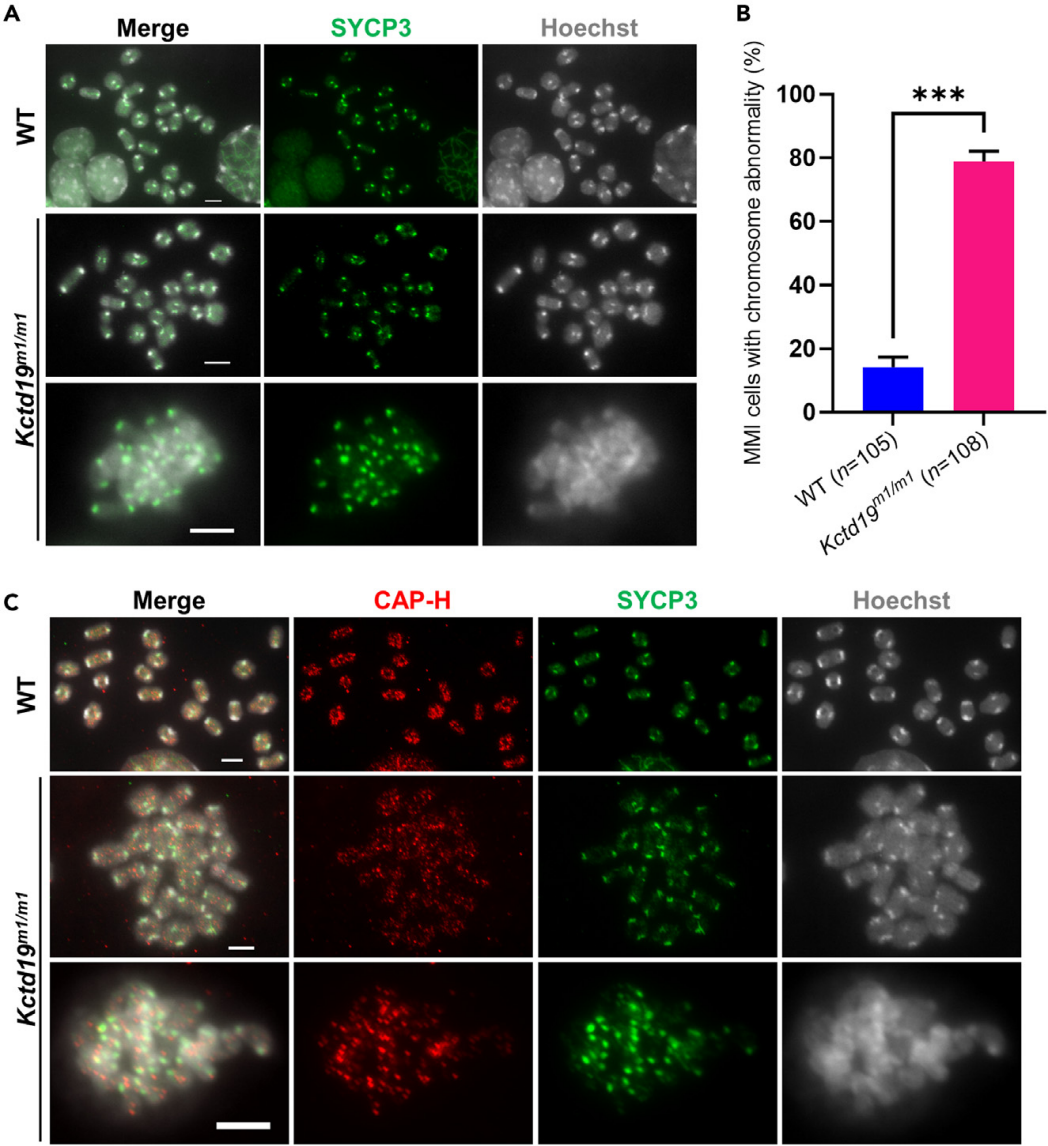
*Kctd19* mutant mice show abnormal individualization of meiotic metaphase I chromosomes (A) Immunofluorescence staining of metaphase I chromosome spreads from WT and *Kctd19*^*m1/m1*^ testes with an antibody against SYCP3 (green). The chromosomes were stained with Hoechst 33342 (gray). In MMI of WT mice, most of the bivalents have a distinct outline and can be easily identified, while in MMI of mutant mice, most of the bivalents are clustered together and difficult to be distinguished individually. Scale bars indicate 5 μm. (B) Proportion of meiotic metaphase I (MMI) spermatocytes with clustered chromosomes in (A). *n*, the number of MMI spermatocytes scored from three mice per genotype. The significance was determined via unpaired Student’s *t* test. ∗∗∗p < 0.001. (C) Immunofluorescence staining of MMI chromosome spreads from WT and *Kctd19*^*m1/m1*^ testes with antibodies against CAP-H (red) and SYCP3 (green). The chromosomes were stained with Hoechst 33342 (gray). Scale bars indicate 5 μm.

To investigate the causes of MMI arrest in *Kctd19*-mutant mice, we performed immunostaining on metaphase I chromosome spreads from WT and *Kctd19* ^*m1/m1*^ testes (Figure 4). SYCP3 was used to identify MMI spermatocytes as granular SYCP3 signals specifically mark centromeres of MMI chromosomes,^26^^,^^27^ and Hoechst was used to mark chromosomes. In 85.71% (90 in 105 from 3 mice, Figure 4B) of MMI spermatocytes from WT testes, bivalent chromosomes were well separated, and almost each bivalent chromosome had a distinct outline that could be distinguished easily (Figure 4A, the first row). In *Kctd19* ^*m1/m1*^ testes, only 20.37% (22 in 108 from 3 mice, Figure 4B) of MMI spermatocytes exhibited bivalent chromosomes with distinct outlines (Figure 4A, the second row), and bivalent chromosomes in the major remaining MMI spermatocytes clustered together, making it impossible to distinguish each individual bivalent (Figure 4A, the third row), indicating that the individualization of metaphase I bivalent chromosomes in *Kctd19* ^*m1/m1*^ spermatocytes was compromised.

Furthermore, we immunostained CAP-H, a subunit of condensin I, which is an essential component of the metaphase chromosome scaffold,^28^ on metaphase I chromosome spreads from WT and *Kctd19* ^*m1/m1*^ testes. We found that CAP-H signals were distributed along the axial regions of well-individualized bivalent chromosomes from both WT and *Kctd19* ^*m1/m1*^ MMI spermatocytes (Figure 4C, the first and second rows), similar to previous observations on mitotic metaphase chromosomes^29^ and on MMI chromosomes of mouse oocytes.^30^ However, on clustered chromosomes from *Kctd19* ^*m1/m1*^ MMI spermatocytes, the CAP-H distribution was disordered (Figure 4C, the third row), indicating that the chromosome scaffold was impaired in these abnormal chromosomes. Together, these results suggested that MMI chromosome individualization was largely damaged in *Kctd19* mutant mice.

### *Kctd19* mutations led to the loss of KCTD19 proteins in testes

**Figure 5:**
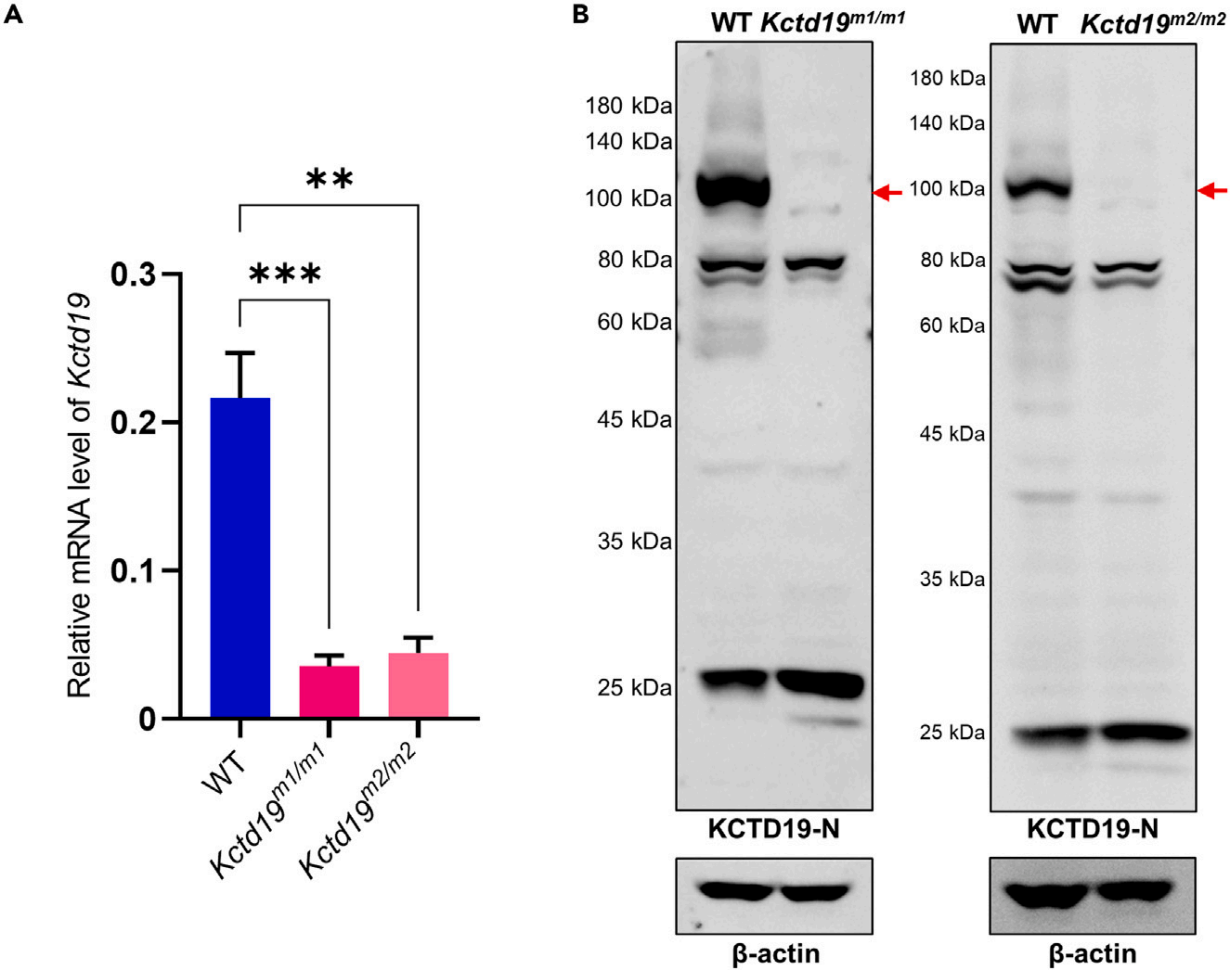
*Kctd19* mutations result in decreased *Kctd19* mRNA levels and loss of KCTD19 protein in testes (A) Quantitative real-time PCR analysis of *Kctd19* mRNA levels in testes from adult WT, *Kctd19*^*m1/m1*^ and *Kctd19*^*m2/m2*^ mice. *Actb* was used as the internal control. Data are from three experiments (three mice per genotype in total). The significance was determined via one-way ANOVA followed by Dunnett’s test. ∗∗∗p < 0.001, ∗∗p < 0.01. (B) Western blotting with testis lysates from adult WT, *Kctd19*^*m1/m1*^ and *Kctd19*^*m2/m2*^ mice using an anti-KCTD19-N antibody. Red arrows indicate the target proteins. β-actin was used as the loading control.

Next, we wondered how these *Kctd19* mutations functioned in mouse testes. We first performed qPCR to measure *Kctd19* mRNA levels in the mutant mice. The mRNA levels of *Kctd19* in the testes from *Kctd19* ^*m1/m1*^ and *Kctd19* ^*m2/m2*^ mice were decreased to approximately 20% of those in WT mice (Figure 5A). Then, western blotting using an antibody against the 1–300 amino acids of KCTD19 (anti-KCTD19-N antibody) was performed with testis lysates from adult WT, *Kctd19* ^*m1/m1*^ and *Kctd19* ^*m2/m2*^ mice. Neither KCTD19 protein nor the predicted KCTD19 truncated proteins were detected in the testes of the *Kctd19* mutant mouse lines (Figure 5B). The loss of KCTD19 protein was also confirmed by western blotting and immunofluorescence staining with another antibody against the 355–753 amino acids of KCTD19 (anti-KCTD19-M antibody) in the testes of both mutant mouse lines (Figure S6). It should be noted that a few supernumerary bands were detected in western blotting, probably because the specificity of these two customized polyclonal anti-KCTD19 antibodies were not perfect when used in western blotting of testis lysates. The anti-KCTD19-M antibody was validated by western blotting and immunofluorescence staining (Figure S7). In western blotting, the antibody successfully detected wild-type and mutated human KCTD19 proteins fused to EGFP in HEK293T cell lysates (Figure S7A). Immunostaining of testicular sections from adult WT mice showed positive staining with the anti-KCTD19-M antibody, while IgG, Omission and negative controls showed no staining signals in the germ cells (Figure S7B). These results indicate that the anti-KCTD19-M antibody is specific and suitable for detecting KCTD19 protein and confirm the loss of KCTD19 protein in the testes of both mutant mouse lines.

Furthermore, we performed immunofluorescence staining on testicular sections from P2034, who carried homozygous M2(c.1897C>T, p.Gln633∗) and a control man, using the anti-KCTD19-M antibody. In the control, KCTD19-positive cells were observed in the seminiferous tubules, as reported previously in mouse testes.^18^^,^^20^ However, KCTD19-positive cells were not detected in the seminiferous tubules from P2034 (Figure S8), similar to the staining results in *Kctd19* mutant mice (Figure S6).

Taken together, we have demonstrated that *Kctd19* mutations in mice severely decreased its mRNA abundance and completely abolished its proteins, and the same loss-of-function effect was observed in one affected individual carrying homozygous *KCTD19* variants. Notably the predicted truncated KCTD19 protein (p.Gln633∗) was detected in cultured cells transfected with vector fused with the corresponding *KCTD19* mutant coding sequence (Figures S3 and S7A). Because of the unavailability of testicular sections from other affected individuals, we could not determine whether the predicted truncated KCTD19 proteins were present in their testes. These results indicate that the effects of a specific variant on the corresponding protein may be different between testes *in vivo* and cultured cells *in vitro*.

## Discussion

Our study identified three homozygous *KCTD19* variants from three azoospermic brothers born of a consanguineous marriage, and two sporadic Chinese NOA-affected individuals exhibiting complete MMI arrest. *Kctd19* mutant mouse models were generated and recapitulated the meiotic defects observed in affected individuals due to severe disrupted individualization of MMI chromosomes. Further analysis showed that the *Kctd19* mutations led to extremely low *Kctd19* mRNA levels and undetectable proteins in the testes of the mutant mice, as well as in the testis of one NOA-affected individual. Taken together, these results illustrate the pathogenicity of *KCTD19* loss-of-function variants in NOA-affected individuals and confirm a conserved and vital role for KCTD19 in MMI chromosome individualization.

Human KCTD genes have emerged in association with neurodevelopmental, neuropsychiatric, and neurodegenerative disorders, as well as several types of cancer.^10^ However, studies on KCTD genes in human Mendelian diseases are still very limited. Pathogenic variants in only *KCTD1* and *KCTD7* have been well established in monogenic Mendelian diseases in humans. Several missense variants in *KCTD1* have been reported to cause scalp-ear-nipple syndrome in an autosomal dominant pattern.^11^ Nonsense as well as missense variants in *KCTD7* cause progressive myoclonic epilepsy with or without intracellular inclusions in an autosomal recessive pattern.^13^^,^^14^^,^^31^^,^^32^ Our study provides genetic and functional evidence that loss-of-function variants in *KCTD19* cause autosomal recessive spermatogenic failure and male infertility, extending the current phenotypic spectrum associated with KCTD gene variants.

MMI arrest has been considered an important cause of NOA-affected individuals,^33^ while only a few genes, such as *HFM1* and *RAD51AP2* have been reported to cause MMI arrest in humans.^34^^,^^35^ Here, we provide both genetic and functional evidence that homozygous variants in *KCTD19* are strongly associated with MMI arrest in males. *Kctd19* mutant mice mimicking these identified *KCTD19* variants, recapitulated the MMI arrest phenotype of individuals carrying homozygous *KCTD19* variants and exhibited the same phenotype as recently reported for *Kctd19* knockout mice.^18^^,^^19^^,^^20^^,^^36^ Further detection of mutation effects in *Kctd19* mutant mouse testes or testicular sections from P2034 showed a complete loss of KCTD19 protein, confirming the pathogenicity of these *KCTD19* variants.

Truncating variants, such as nonsense variants and frameshift variants, can cause nonsense-mediated mRNA decay with no protein expression^37^ or the production of truncated proteins.^38^ Here, the presence of mutated *KCTD19* mRNAs was detected in the blood using nested PCR from the affected individuals, and all three mutated KCTD19 proteins were also detected by western blotting in cultured cells transfected with vectors bearing the mutant coding sequence, suggesting that the predicted truncated proteins may be expressed in the affected individuals. However, in testes from *Kctd19* ^*m1/m1*^ and *Kctd19* ^*m2/m2*^ mice, which mimicked the variants identified in our affected individuals, very low *Kctd19* mRNA levels and complete loss of KCTD19 proteins were detected. These findings are consistent with the absence of KCTD19 signals in the testicular sections of P2034, indicating that no truncated proteins existed in this affected individual. These results highlighted the need for *in vivo* validation of the effects of candidate pathogenic variants identified in affected individuals. Obviously, the expression of truncated KCTD19 proteins in HEK293T cells transfected with mutated KCTD19-expressing vectors does not indicate their definite expression *in vivo*. Thus, when variant effects on mRNA or protein levels cannot be measured in affected individuals’ samples, animal models that mimic these variants or some more convincing *in vitro* models may be better approaches.

Through careful analysis of the cause of MMI arrest in *Kctd19* mutant mice, we demonstrated that KCTD19 was involved in MMI chromosome individualization, using the metaphase chromosome spreading method and SYCP3 immunofluorescence staining of the spreads. Deficiency of KCTD19 in mice resulted in abnormal individualization of MMI bivalent chromosomes, and this phenotype had never been found in any mutant or knockout mice that exhibited MMI arrest. Notably, although the same metaphase I chromosome spreading method was performed, former studies didn’t find this abnormal phenotype in *Kctd19* knockout mice,^18^^,^^20^ perhaps because those clumped chromosomes were not considered MMI chromosomes after Giemsa staining. Metaphase chromosome formation is a highly complicated process with numerous aspects that remain unclear and many details remain to be elucidated.^39^ Abnormal metaphase chromosome formation usually results from depletion of chromosome scaffold proteins such as condensin and topoisomerase IIα (TOP2A).^40^^,^^41^ However, according to current knowledge, KCTD19 is more likely a transcriptional associated protein than a chromosome scaffold protein.^18^^,^^36^ Recently, it was reported that KCTD19 interacts with ZFP541, DNTTIP1, and HDAC1/2 to form an HDAC complex during mouse meiosis, which functions as a critical transcriptional repressor to maintain the suppression status of a broad range of genes, including genes associated with meiotic DNA double-strand break formation, transcriptional regulation, and covalent chromatin modification, thus ensuring the progression of meiosis I.^18^^,^^42^ In addition, no known chromosome scaffold proteins have yet been identified to interact with KCTD19.^18^^,^^20^ Based on these findings, we assumed that this novel phenotype in MMI chromosome individualization was highly related to the drastic changes in the transcriptome in KCTD19-deficient spermatocytes. In the future, we will study how KCTD19 functions in metaphase chromosome formation.

Moreover, we found that *KCTD19* variants caused the loss of KCTD19 protein, and *Kctd19* mutant mice recapitulated the MMI arrest phenotype of men with homozygous *KCTD19* variants, which suggests a conserved function of KCTD19 between humans and mice. It can be inferred that the function of the aforementioned HDAC complex was also disrupted due to *KCTD19* variants in the affected individuals. Thus, our study indicates that this HDAC complex is an important regulator of meiosis in humans. It would be intriguing to explore whether potential pathogenic variants in other members of this HDAC complex are also associated with male infertility.

It is worth noting that three loss-of-function variants in *KCTD19* were identified in different ethnic populations. Two *KCTD19* variants (p.Tyr68Thrfs∗17 and p.Gln633∗) were found in two of 102 sporadic Chinese NOA individuals with meiotic arrest. The third *KCTD19* variant (p.Gln669∗) was found in one of 50 Pakistani consanguineous families with at least two infertile siblings in each. Given that the 17-year-old brother Ⅳ:7 from Family-01 also carries homozygous *KCTD19* variants (p.Gln669∗), it is recommended that he should undergo andrology tests in adulthood. These results suggest that pathogenic variants in *KCTD19* are closely linked with male infertility in different ethnic groups and have important implications for genetic counseling.

In conclusion, our study based on NOA affected individuals and mutant mouse models demonstrate that homozygous loss-of-function variants in *KCTD19* cause meiotic arrest at MMI and male infertility in mice and humans. Our findings provide further evidence that meiotic arrest is often of monogenic origin^43^ and highlight the essential and conserved role of *KCTD19* in meiosis metaphase chromosome individualization. *KCTD19* can be used as a genetic screening marker for male infertility and to assess the chances of successful sperm retrieval prior to testicular biopsy.

### Limitations of the study

In this study, we have successfully identified pathogenic variants of *KCTD19* in two infertile Chinese men and in a Pakistani family consisting of three infertile brothers. The pathogenicity of these *KCTD19* variants was validated in mutant mouse models. However, there are some limitations of this study that should be addressed in future research efforts. Firstly, the sample size in our study was small, thus, the findings may not be generalized to other populations. Further research studies involving a larger cohort of infertile men are required to accurately determine the clinical significance and implications of *KCTD19* variants in male infertility. Secondly, although, we have explored the novel role of KCTD19 in MMI chromosome individualization, our study did not identify any possible chromosome scaffold proteins that could be responsible for the phenotype observed in KCTD19-deficient spermatocytes. Therefore, more research is needed to comprehensively elucidate the functions of KCTD19 in metaphase chromosome formation.

## STAR★Methods

### Key resources table

**Table:** 

REAGENT or RESOURCE	SOURCE	IDENTIFIER
**Antibodies**		

H3S10p (IF, 1:5000)	Santa Cruz Biotechnology	Cat# sc-8656-R; RRID: AB_653256
SYCP3 (IF, 1:200)	Abcam	Cat# ab97672; RRID: AB_10678841
CAP-H (IF, 1:100)	Proteintech	Cat# 11515-1-AP; RRID: AB_2150003
KCTD19-M (IF, 1:100; WB, 1:2000)	This study	Customized production by ABclonal (China)
KCTD19-N (WB, 1:2000), not suitable for IF staining	This study	Customized production by ABclonal (China)
GFP (WB, 1:3000)	Abmart	Cat# M20004; RRID: AB_2619674
β-actin (WB, 1:6000)	Abcam	Cat# ab8227; RRID: AB_2305186
GAPDH (WB, 1:2000)	Millipore	Cat# MAB374; RRID: AB_2107445
Goat Anti-Mouse IgG1, Alexa Fluor 488 Conjugated (IF, 1:100)	Thermo Fisher Scientific	Cat# A-21121; RRID: AB_2535764
Donkey Anti-Rabbit IgG (H+L), Alexa Fluor 555 Conjugated (IF, 1:200)	Thermo Fisher Scientific	Cat# A-31572; RRID: AB_162543
HRP Donkey anti-rabbit IgG (WB, 1:10000)	BioLegend	Cat# 406401; RRID: AB_2099368
HRP Goat anti-mouse IgG (WB, 1:10000)	BioLegend	Cat# 405306; RRID: AB_315009

**Bacterial and virus strains**		

Trans5α Chemically Competent Cell	TransGen Biotech	Cat# CD201

**Biological samples**		

Paraffin-embedded human testicular tissue blocks	This study	N/A
Frozen human peripheral blood samples	This study	N/A

**Chemicals, peptides, and recombinant proteins**		

RNAiso Plus	TaKaRa	Cat# 9109
Bouin’s solution	Sigma	Cat# HT10132
Triton X-100	Sigma	Cat# T9284
Lectin PNA, Alexa Fluor 568 Conjugate	Thermo Fisher Scientific	Cat# L32458
Normal rabbit IgG	Cell Signaling Technology	Cat# 2729S
VECTASHIELD Antifade Mounting Medium	Vector Laboratories	Cat# H-1000
Hoechst 33342	Invitrogen	Cat# H3570
Dulbecco’s Modified Eagle Medium with high glucose	HyClone	Cat# SH30022.01
Fetal Bovine Serum	Gibco	Cat# 16000044
Penicillin-Streptomycin	Gibco	Cat# 15140122
PMSF Protease Inhibitor	Thermo Fisher Scientific	Cat# 36978

**Critical commercial assays**		

FlexiGene DNA Kit	QIAGEN	Cat# 51206
TIANScript II RT kit	TIANGEN	Cat# KR107
PrimeSTAR® HS DNA Polymerase	TaKaRa	Cat# R044A
FastStart Universal SYBR Green Master Mix	Roche	Cat# 04913850001
Lipofectamine® 3000	Thermo Fisher Scientific	Cat# L3000008

**Oligonucleotides**		

sgRNAs and primers used in this study are listed in Table S3	This study	N/A

**Experimental models: Cell lines**		

HEK293T	ATCC	Cat# CRL-3216; RRID: CVCL_0063

**Experimental models: Organisms/strains**		

Mouse: C57BL/6N	Beijing Vital River Laboratory Animal Technology Co.	Strain Code: 213
Mouse: ICR	Beijing Vital River Laboratory Animal Technology Co.	Strain Code: 201
Mouse: *Kctd19*^*m1/m1*^	This paper	N/A
Mouse: *Kctd19*^*m2/m2*^	This paper	N/A

**Software and algorithms**		

Sorting Intolerant From Tolerant (SIFT)	SIFT	RRID:SCR_012813 https://sift.bii.a-star.edu.sg/
PolyPhen-2	PolyPhen-2	RRID:SCR_013189 http://genetics.bwh.harvard.edu/pph2/
MutationTaster	MutationTaster	RRID:SCR_010777 http://www.mutationtaster.org/
MutationAssessor	MutationAssessor	RRID:SCR_005762 https://mutationassessor.org/
FATHMM	FATHMM	http://fathmm.biocompute.org.uk/
GERP++	GERP++	RRID:SCR_000563 http://mendel.stanford.edu/SidowLab/downloads/gerp/
SiPhy	SiPhy	RRID:SCR_000564 https://portals.broadinstitute.org/genome_bio/siphy/
PedMiner	PedMiner	https://mcg.ustc.edu.cn/bsc/pedminer/
BCFtools/RoH	BCFtools/RoH	https://samtools.github.io/bcftools/howtos/roh-calling.html
NIS-elements Basic Research	Nikon	RRID: SCR_002776
CellSens	Olympus	RRID: SCR_014551
GraphPad Prism	GraphPad	RRID: SCR_002798

### Resource availability

#### Lead contact

Further information and requests for resources and reagents should be directed to and will be fulfilled by the Lead Contact, Dr. Qinghua Shi (qshi@ustc.edu.cn).

#### Materials availability

All unique materials generated in this study will be made available upon request to the lead contact.

### Experimental model and subject details

#### Clinical samples

In this study, we recruited 50 consanguineous Pakistani families, each with at least two infertile siblings, and 102 infertile Chinese men diagnosed with NOA due to meiotic arrest. After we obtained informed consent, the participants donated blood samples and testicular tissues for this research. Routine semen analyses were performed according to the WHO guidelines.^44^ Serum levels of reproductive hormones were measured in the local laboratories. This study was approved by the institutional ethics committee of the University of Science and Technology of China (USTC) with approval number 2019-KY-168.

#### Cell line and transfection

Wild-type (WT) and mutated (MT1, MT2 and MT3) *KCTD19* coding sequences were fused to the C-terminus of EGFP and cloned into pEGFP-N1 vectors. HEK293T cells (ATCC, CRL-3216, USA) were cultured in high-glucose DMEM (Hy-Clone, SH30022.01, USA) supplemented with 10% FBS (GIBCO, 15140122, USA), 100 U/ml penicillin, and 100 mg/ml streptomycin (GIBCO, 16000044, USA) in 24-well plates. All the cultures were maintained in 5% CO_2_ at 37°C. Cells were passaged 2-3 times after thawing and then transfected with vectors using Lipofectamine 3000 (Invitrogen, L3000008, USA) according to the manufacturer’s instructions. After 36 h of transfection, cells were collected and lysed in 1 × SDS sample buffer (300 mM Tris, pH 7.4, 2% SDS, 15% glycerol, 0.1% bromophenol blue, and 5 mM dithiothreitol). Cell lysates were denatured at 100°C for 10 min and then analyzed by Western blotting.

#### Mice

*Kctd19* (GenBank: NM_001301173.1) mutant mice were generated by CRISPR/Cas9 technology as previously described.^35^ Briefly, Cas9 mRNAs and two small guide RNAs (sgRNAs) targeting exon 2 or one single guide RNA targeting exon 12 were co-injected into C57BL/6 zygotes, followed by embryo transfer into pseudo-pregnant ICR females. Newborn mice were genotyped by PCR and Sanger sequencing. The founder mice that carried the mutant *Kctd19* allele(s) were backcrossed onto the C57BL/6 background. Homozygous mutants obtained from the F2 generation were used for subsequent experiments. All mice were maintained under specific pathogen-free conditions in the laboratory animal center of USTC. All experiments involving animals were approved by the institutional animal ethics committee of USTC. The sgRNA sequences and genotyping primers are listed in Table S3.

### Method details

#### Whole exome sequencing, variant filtration and validation

Total genomic DNA was extracted from the peripheral blood of individuals using a FlexiGene DNA Kit (QIAGEN, 51206, Germany) according to the manufacturer’s instructions. AIExome Enrichment Kit V1 (iGeneTech, Beijing, China)-captured libraries were constructed as instructed by the manufacturer. Sequencing was carried out on a HiSeq2000 platform (Illumina, San Diego, CA, USA). Clean reads were aligned to the human genome reference assembly (hg19) using Burrows–Wheeler Aligner (BWA) with default parameters.^45^ Then, Picard software (http://picard.sourceforge.net/) was employed to remove polymerase chain reaction (PCR) duplicates. DNA sequence variants were called using the Genome Analysis Toolkit HaplotypeCaller (http://www.broadinstitute.org/gatk/). Variants were annotated using ANNOVAR.^46^

After performing WES of the whole study cohort, only those individuals who were found to carry biallelic *KCTD19* variants were included in our analysis, as our study aimed to investigate the relationship between *KCTD19* variants and human fertility. Specifically, we selected two sporadic NOA-affected individuals (P7864 and P2034), as well as a consanguineous family (Family-01). The variant filtration processes of their WES data are as follows: Candidate variant filtration was performed in a stepwise manner as we previously described.^47^^,^^48^^,^^49^ In brief, for the consanguineous Pakistani family, linkage analysis was performed using PedMiner^50^ and four regions were identified with logarithm of the odds scores >0. Variants within linkage regions and following recessive inheritance were kept for further screening. For the two sporadic NOA-affected individuals (P7864 and P2034), runs of homozygosity (RoH) were first detected using BCFtools/RoH^51^ and RoH regions >1.5 Mb were used to calculate the FROH value to measure the inbreeding coefficients using our inhouse scripts. They were found to have an FROH >0.01 and were thought to be offspring of consanguineous marriages.^52^ Thus, homozygous variants within RoH regions were kept for further screening. Variants meeting the following conditions were given preference: (1) variants potentially affecting protein sequence (nonsense, missense, splice-site variants, and coding indels); (2) variants with minor allele frequencies (MAF) <0.01 in 1000 Genomes project, ESP6500, or GnomAD database; (3) loss-of-function variants or potentially deleterious missense variants predicted by 7 software programs including Sorting Intolerant From Tolerant (SIFT),^53^ PolyPhen-2,^54^ MutationTaster,^55^ MutationAssessor,^56^ FATHMM,^57^ GERP++,^58^ and SiPhy^59^ for predicting the pathogenicity of variants. Variants predicted to be deleterious by at least half of the programs covering the variants were kept for further analysis; (4) Variants within genes expressed in testes. Finally, variants within genes dispensable for spermatogenesis based on the MGI database,^60^ FertilityOnline database^61^ or literature search were excluded (Table S4).

After filtration, the identified candidate variants that may cause spermatogenesis arrest were subsequently verified by Sanger sequencing, and the primer sequences used are shown in Table S3.

#### RNA extraction, PCR and quantitative real-time PCR

Total RNA was extracted with RNAiso Plus reagents (TaKaRa, 9109, Japan) followed by cDNA synthesis using the TIANScript II RT kit (TIANGEN, KR107, Japan) according to the manufacturer’s protocol. PrimeSTAR HS DNA polymerase (TaKaRa, R044A, Japan) was used for PCR. Nested PCR was carried out for the detection of *KCTD19* mRNA in blood samples, where a primary PCR mixture was used as the template for secondary PCR with nested primers. For the primary PCR, the following cycle conditions were used: 5 min at 98°C, followed by 40 cycles of 15 s at 98°C, 15 s at 60°C, and 60 s at 72°C. For nested PCR, the following cycle conditions were used: 5 min at 98°C, followed by 38 cycles of 15 s at 98°C, 15 s at 60°C, and 30 s at 72°C. Quantitative real-time PCR (qPCR) was performed with FastStart Universal SYBR Green Master Mix (Roche, 04913850001, Switzerland) using a StepOne Real-Time PCR System (Applied Biosystems, USA) as previously reported,^49^ and relative mRNA levels were calculated by normalization to *Actb*. Primer sequences are provided in Table S3.

#### Histological analysis and immunofluorescence staining

Hematoxylin and eosin (H&E) staining of testicular and epididymal sections was performed as we previously described.^47^^,^^62^ Briefly, testicular tissues were fixed in Bouin’s solution or 4% PFA overnight, embedded in paraffin and sectioned at 5 μm thickness. Slides were deparaffinized by xylene, rehydrated with gradient ethanol and then stained with hematoxylin and eosin (H&E). Immunofluorescence staining of the testicular sections was conducted as we previously described.^62^^,^^63^ Images were captured and analyzed using a Nikon ECLIPSE 80i microscope with NIS-elements BR software (Japan) or an Olympus BX53 microscope with cellSens imaging software (Japan). The antibodies used are listed in key resources table.

#### Generation of polyclonal anti-KCTD19 antibodies

KCTD19 polyclonal antibodies were generated in rabbits using amino acids 1-300 (KCTD19-N) and 355-753 (KCTD19-M) of mouse KCTD19 (UniProt accession no. Q562E2) as antigens by ABclonal Biotechnology. Briefly, gene fragments encoding the two epitopes were cloned into pET-28a expression vectors, and the His-tagged fusion proteins were expressed in *Escherichia coli*. The purified recombinant proteins were then used as antigens for producing polyclonal antisera in New Zealand rabbits.

#### Western blotting

Testes from adult mice were homogenized in lysis buffer (50 mM Tris, pH 7.5, 150 mM NaCl, 0.5% Triton X-100, 2.5 mM EDTA, 1% sodium deoxycholate and 0.1% sodium dodecyl sulfate) containing a 1 × PMSF protease inhibitor mixture (Thermo Scientific, 36978, USA). Western blotting was performed as previously described.^64^ Briefly, the lysates were denatured at 100°C for 10 min and separated by SDS–polyacrylamide gel electrophoresis, followed by transferring the proteins to immobilon-P membranes (Millipore, IPVH00010, USA) using a vertical electrophoresis and blotting apparatus (Tanon, China). Membranes were blocked in PBST containing 5% nonfat milk at 25°C for 60 min and incubated with primary antibodies diluted in PBST at 4°C overnight. Following incubation with horseradish peroxidase (HRP)-conjugated secondary antibodies diluted in PBST at 25°C for 90 min, the membranes were developed with chemiluminescence substrate by an ImageQuant LAS 4000 imaging system (GE Healthcare, USA). The antibodies used are listed in key resources table.

#### Meiotic metaphase I chromosome spreading and immunofluorescence staining

Mouse meiotic metaphase I chromosome spreads were prepared as we previously described.^64^ Before immunofluorescence staining, slides were heated at 98°C for 20 min in citrate buffer (10 mM sodium citrate and 1 mM citric acid) for antigen retrieval.

Immunofluorescence staining of MMI chromosome spreads was conducted as we previously described.^63^^,^^64^ Images were captured and analyzed using a Nikon ECLIPSE 80i microscope with NIS-elements BR software (Japan). The antibodies used are listed in key resources table.

### Quantification and statistical analysis

Statistical analyses were conducted using GraphPad Prism software (USA). Data are presented as mean ± SEM of at least three independent experiments. Unpaired Student’s *t* test and one-way ANOVA followed by Dunnett's test were used to assess statistical significance. Statistical significance was defined as p < 0.05. All tests and p values are described in the corresponding figure legends and/or results.

## Data Availability

Data: Data reported in this paper will be shared by the lead contact upon request. Code: This paper does not report original code. Additional information: Any additional information required to reanalyze the data reported in this paper is available from the lead contact upon request.
