# Supplementary material for: Loss-of-function variants in KCTD19 cause non-obstructive azoospermia in humans
Source: iScience. 2023 Jun 28;26(7):107193. doi: 10.1016/j.isci.2023.107193 (PMC10362269; doi:10.1016/j.isci.2023.107193)
Supplement: Document S1. Figures S1–S8 and Tables S1–S4 [file mmc1.pdf]

iScience, Volume *26*

Supplemental information

Loss-of-function variants in *KCTD19* cause non-obstructive azoospermia in humans

Junyan Liu, Fazal Rahim, Jianteng Zhou, Suixing Fan, Hanwei Jiang, Changping Yu, Jing Chen, Jianze Xu, Gang Yang, Wasim Shah, Muhammad Zubair, Asad Khan, Yang Li, Basit Shah, Daren Zhao, Furhan Iqbal, Xiaohua Jiang, Tonghang Guo, Peng Xu, Bo Xu, Limin Wu, Hui Ma, Yuanwei Zhang, Huan Zhang, and Qinghua Shi

Figure S1

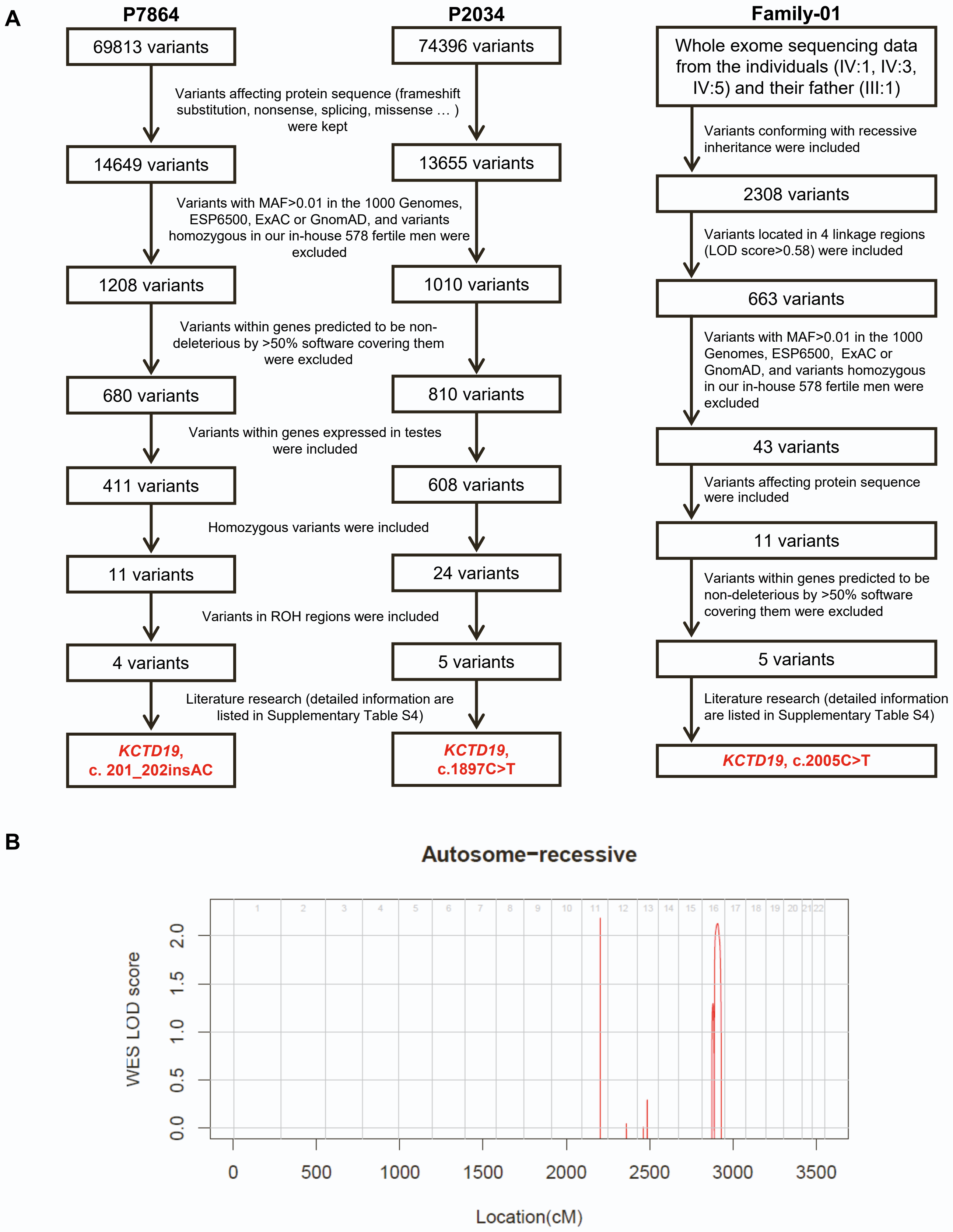

Figure S1. Whole-exome sequencing (WES) data analysis.
(A) The pipeline for whole-exome sequencing data filtration analysis of Family-01, P2034 and P7864. gDNA, genomic DNA. SNVs, single nucleotide variations; MAF, minor allele frequency. ROH, runs of homozygosity.
(B) The linkage region identified using PedMiner for Family-01. LOD, the logarithm of the odds.

Figure S2

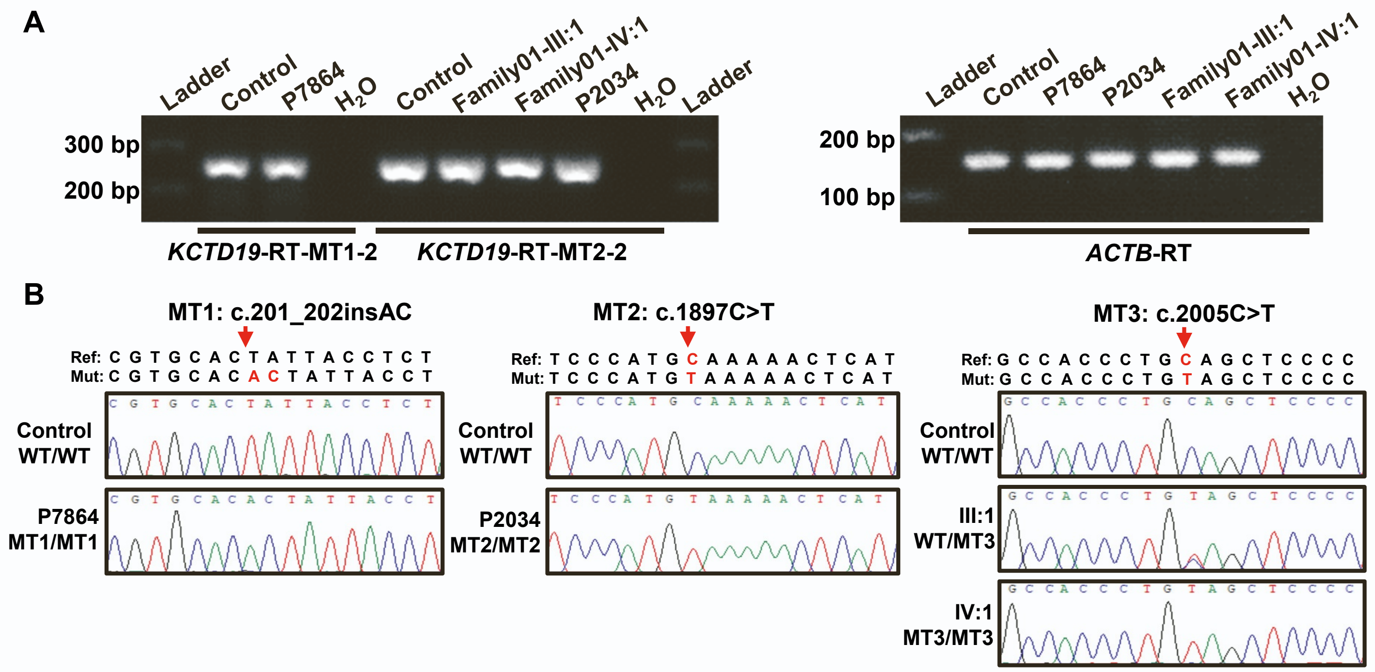

Figure S2. Verification of the *KCTD19* mutations at mRNA level.
(A) Nested RT-PCR of blood samples from a fertile man (control) and other individuals carrying *KCTD19* mutations. The secondary round of PCR for *KCTD19* was amplified by indicated primers. *ACTB* served as the internal control. (B) cDNA sequencing chromatograms show *KCTD19* mutations in all available individuals. Red arrows indicate the mutation sites.

Figure S3

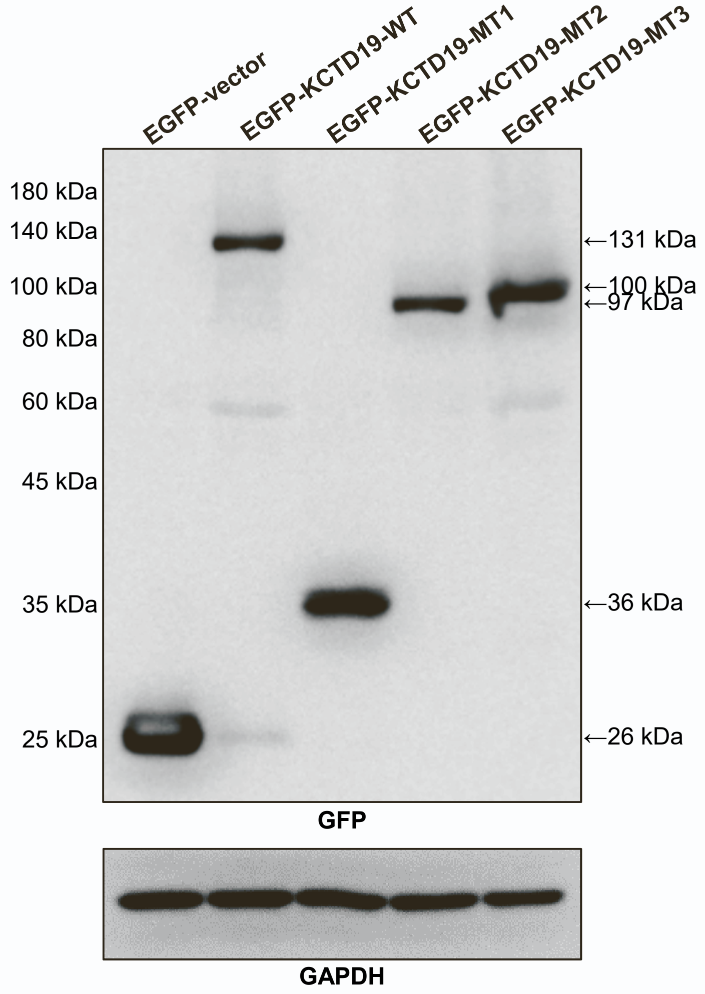

Figure S3. Exogenous expression of mutant KCTD19 proteins in cultured cells.
Western blotting with HEK293T cell lysates after transfection. Wild-type (WT) and mutated (MT1, 2 and 3) human KCTD19 proteins fused to EGFP were detected by an anti-GFP antibody. GAPDH was used as the loading control. Arrows indicate bands corresponding to the fusion proteins and their predicted molecular weights.

Figure S4

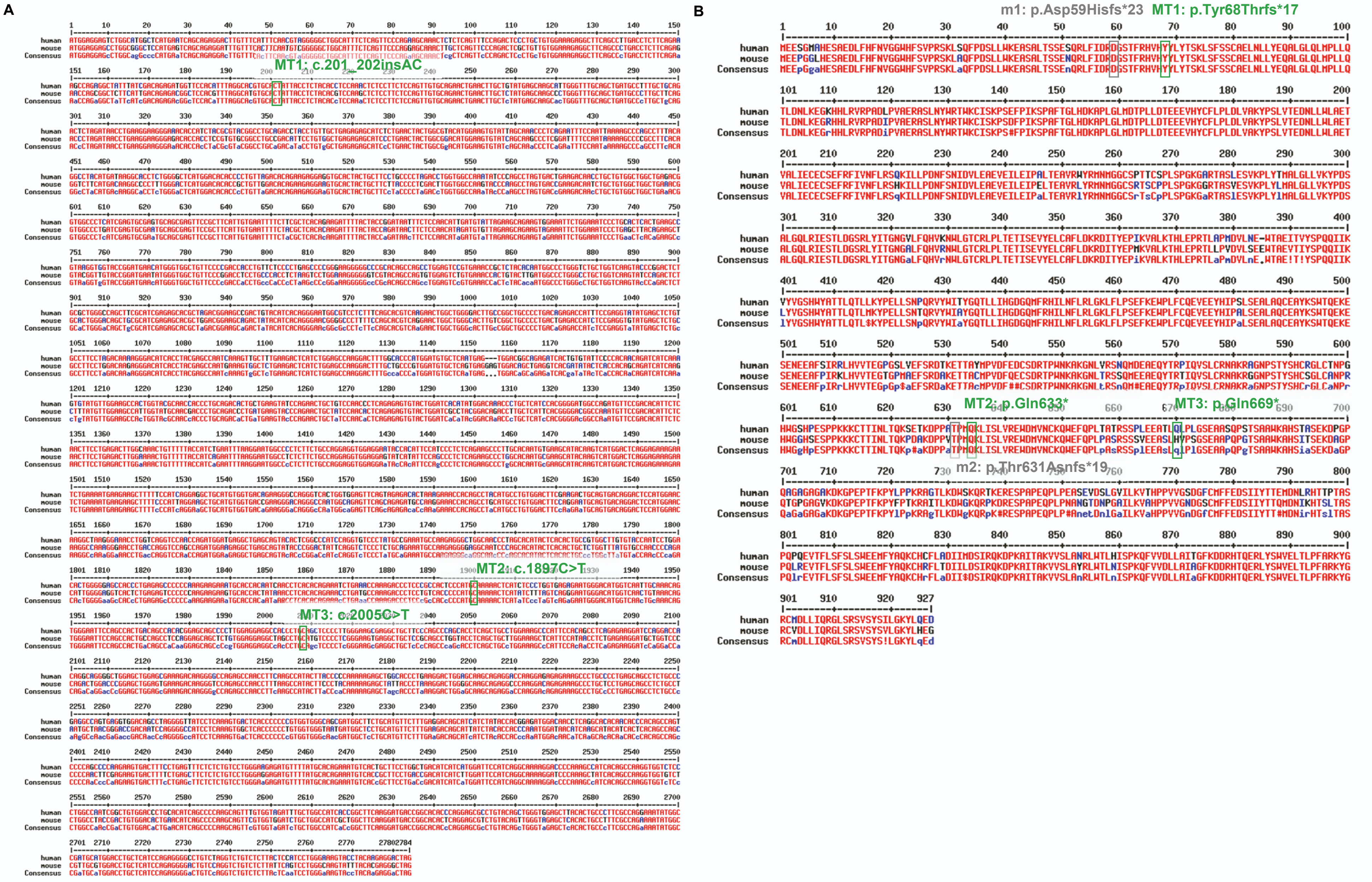

Figure S4. Alignment of human and mouse *KCTD19* coding sequences and protein sequences.
(A) Alignment of human and mouse *KCTD19* coding sequences showing that 87% of nucleotides are identical. Residues that are identical between human and mouse were written in red in the consensus line. Unconserved residues were written in blue in the consensus line. Green boxes and notes indicate human variants identified in this study. The alignment was performed using the online software MultAlin (http://multalin.toulouse.inra.fr/multalin/multalin.html).
(B) Alignment of human and mouse KCTD19 protein sequences showing that 89% of amino acids are identical. Residues that are identical between human and mouse were written in red in the consensus line. Highly similar residues were indicated by red symbols (!, any one of I and V; $, any one of L and M; #, any one of N, D, Q, E, B, and Z). Unconserved residues were written in blue in the consensus line. Green boxes and notes indicate human variants identified in this study. Gray boxes and notes indicate *Kctd19* mutant mouse lines generated in this study. The alignment was performed using the online software MultAlin (http://multalin.toulouse.inra.fr/multalin/multalin.html).

Figure S5

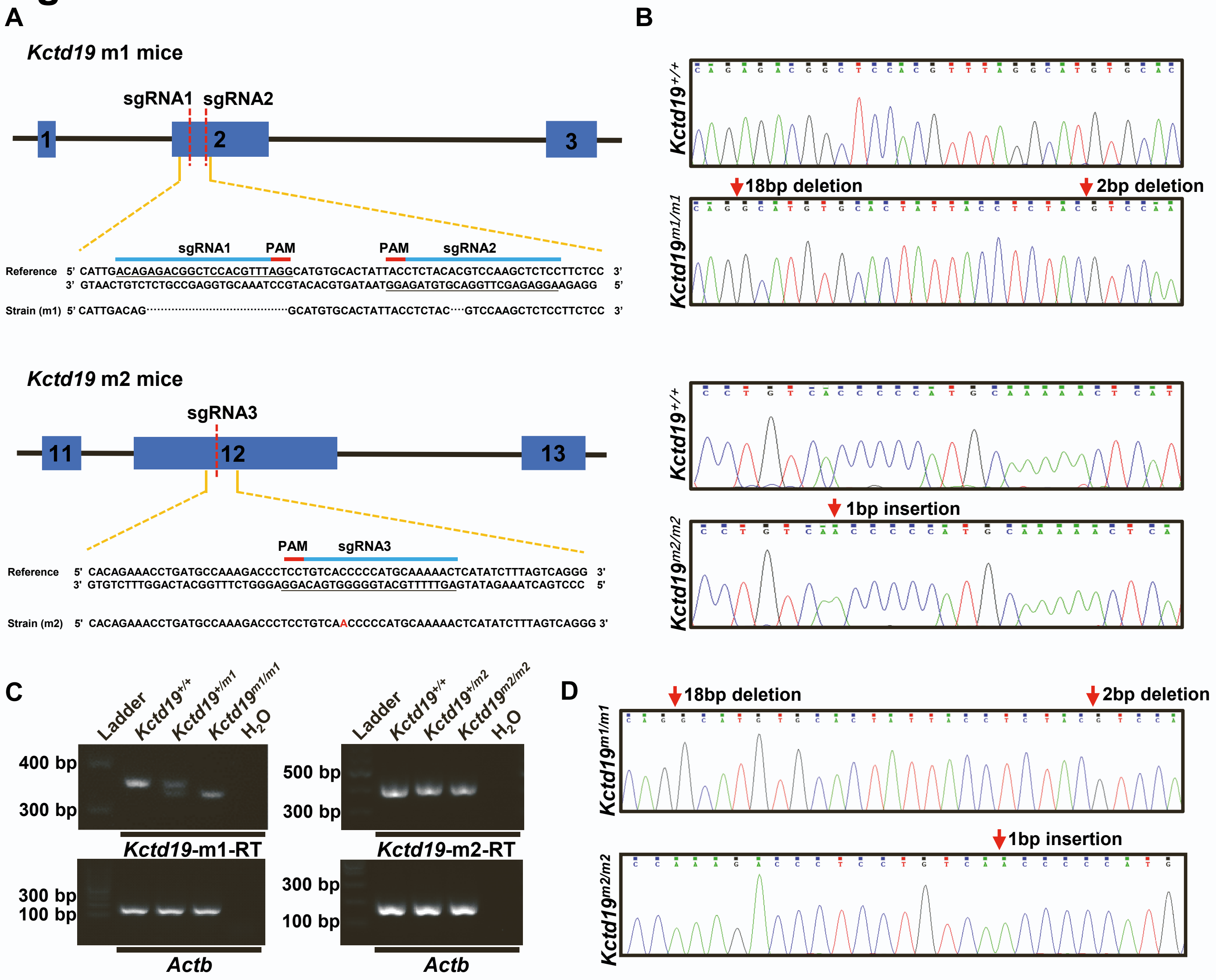

Figure S5. Generation of the *Kctd19*$^{m1/m1}$ and *Kctd19*$^{m2/m2}$ mouse models.
(A) The strategies for the generation of *Kctd19*$^{m1/m1}$ and *Kctd19*$^{m2/m2}$ mice by CRISPR/Cas9 technology. Blue solid squares represent exons. Red dashed lines indicate the location of corresponding single guide RNAs (sgRNAs). Nucleotides with black underlines indicate the sgRNA targeting sequence. Black dotted lines indicate the deleted 18 bp and 2 bp in *Kctd19*$^{m1/m1}$ mice. The nucleotide written in red indicates the inserted 1 bp in *Kctd19*$^{m2/m2}$ mice. PAM, protospacer adjacent motif.
(B) Genomic DNA sequencing chromatograms showing the genomic DNA changes in *Kctd19* in *Kctd19*$^{m1/m1}$ and *Kctd19*$^{m2/m2}$ mice, respectively. Red arrows indicate the mutation sites.
(C) RT-PCR of mouse testis samples. *Kctd19* was amplified by indicated primers. *Actb* served as the internal control.
(D) cDNA sequencing chromatograms confirm the *Kctd19* mutations in mutant mice. Red arrows indicate the mutation sites.

Figure S6

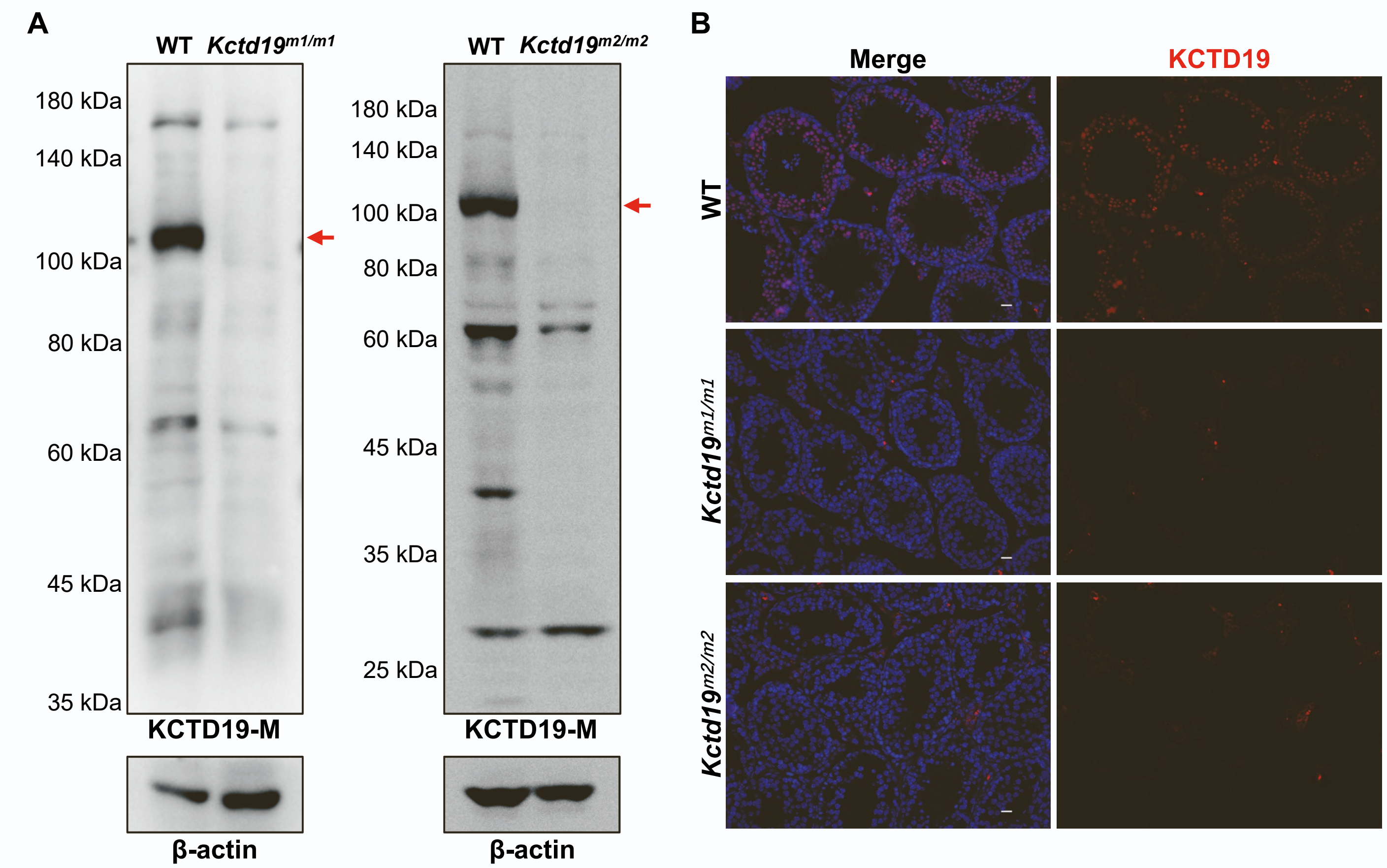

Figure S6. Validation of the loss of KCTD19 proteins in *Kctd19* mutant mice using an anti-KCTD19-M antibody
(A) Western blotting with testis lysates from adult WT, *Kctd19*$^{m1/m1}$ and *Kctd19*$^{m2/m2}$ mice using an anti-KCTD19-M antibody. Red arrows indicate the wild-type KCTD19 proteins. β-actin was used as the loading control.
(B) Immunofluorescence staining of testicular sections from adult WT, *Kctd19*$^{m1/m1}$ and *Kctd19*$^{m2/m2}$ mice using an anti-KCTD19-M antibody (red). The nuclei were stained with Hoechst 33342 (blue). Scale bars indicate 20 μm.

Figure S7

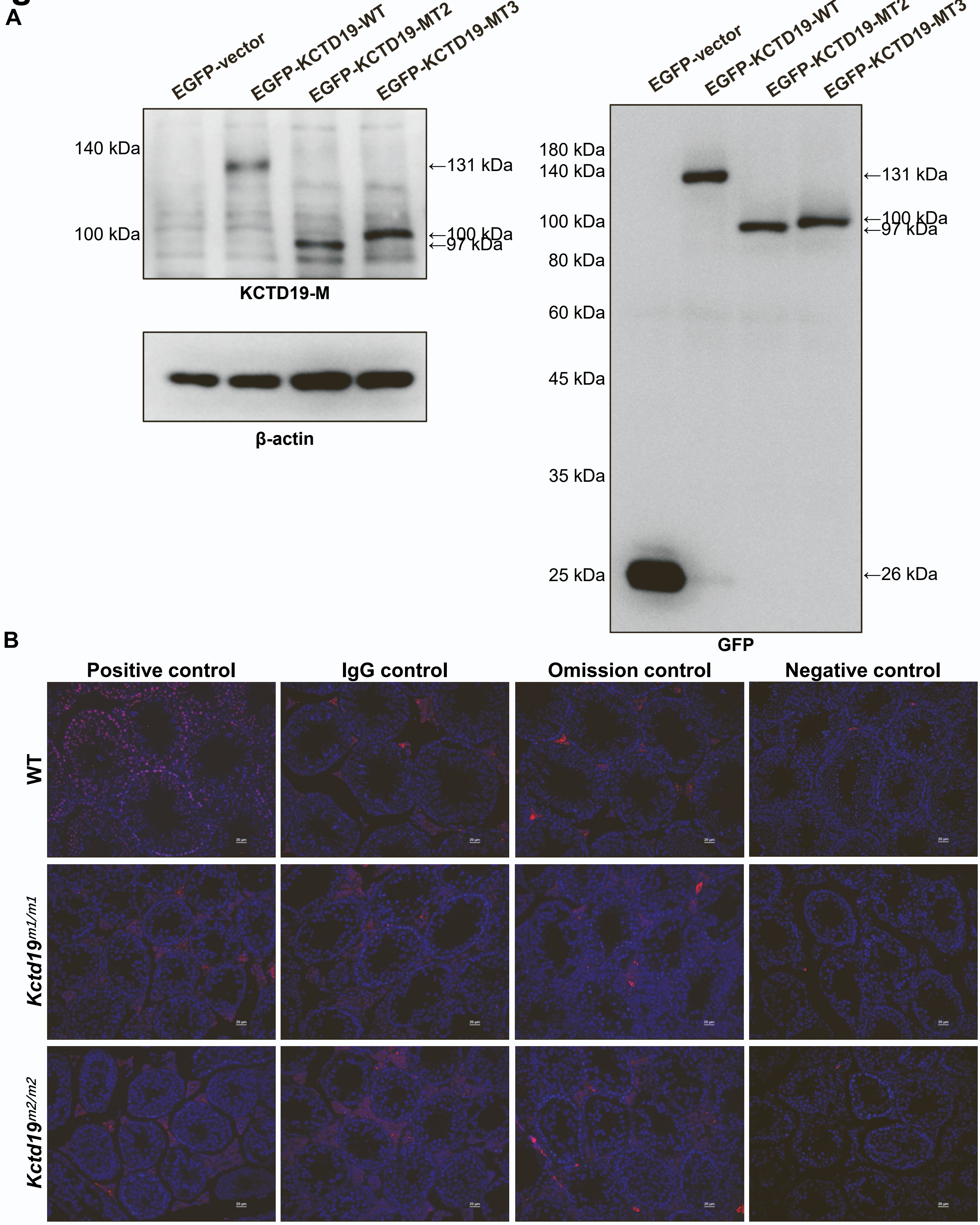

Figure S7. Validation of the anti-KCTD19-M antibody

(A) Western blotting with HEK293T cell lysates after transfection. Wild-type and mutated human KCTD19 proteins fused to EGFP were detected with an anti-KCTD19-M antibody. β-actin was used as the loading control. The GFP antibody was used as a positive control. Arrows indicate bands corresponding to the fusion proteins and their predicted molecular weights.

(B) Immunostaining of testicular sections from adult WT, *Kctd19*$^{m1/m1}$ and *Kctd19*$^{m2/m2}$ mice. Positive control, anti-KCTD19-M antibody (red) and corresponding secondary antibody. IgG control, normal rabbit IgG and corresponding secondary antibody. Omission control, corresponding secondary antibody only. Negative control, without any primary or secondary antibody. The nuclei were stained with Hoechst 33342 (blue). Merged images are shown. Scale bars indicate 20 μm.

Figure S8

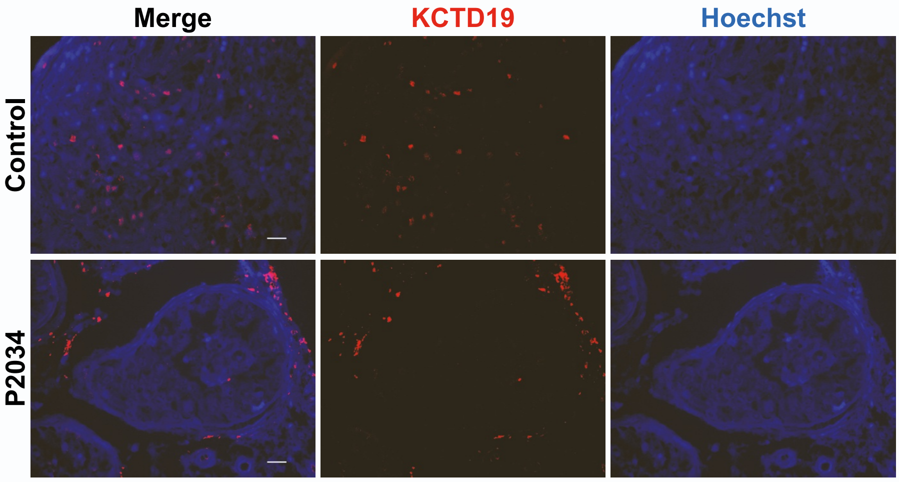

Figure S8. Validation of the loss of KCTD19 protein in P2034 testicular sections
Immunofluorescence staining of testicular sections from the control and P2034 with an anti-KCTD19-M antibody (red). The nuclei were stained with Hoechst 33342 (blue). Scale bars indicate 20 μm.

Table S1. Overview of *KCTD19* variants identified from the affected individuals

Mutation ID	Subjects	Genomic Position on chr16 (bp)	cDNA Change	Protein Change	Genotype	Allele Frequency in Population		
						1KGP	ESP6500	gnomAD
MT1	P7864	67354590	c.201_202insAC	p.Tyr68Thrfs*17	Homozygous	0	0	0
MT2	P2034	67327768	c.1897C>T	p.Gln633*	Homozygous	0	0	0
MT3	Family-01 IV:1, IV:3, IV:5	67327660	c.2005C>T	p.Gln669*	Homozygous	0	0	0

Table S2. Classification of *KCTD19* variants based on ACMG guidelines

Mutation ID	Category	Evidence of pathogenicity	Overall Classification
MT1	PVS1[a]	Predicted to result in loss of function	Likely Pathogenic
	PM2[b]	Absent from population databases	
MT2	PVS1	Predicted to result in loss of function	Likely pathogenic
	PM2	Absent from population databases	
MT3	PVS1	Predicted to result in loss of function	Pathogenic
	PM2	Absent from population databases	
	PP1[c]	Cosegregation with disease in multiple affected family members	

a, PVS1: Pathogenic Very Strong; b, PM: Pathogenic Moderate; c, PP: Pathogenic Supporting.

Table S3. The sgRNAs and primers used in this study

**sgRNAs used for generation of *Kctd19* mutant mice**		
**sgRNA**	**Sequence (5’-3’)**	
mKctd19-m1-sgRNA1	ACAGAGACGGCTCCACGTTT	
mKctd19-m1-sgRNA2	AGGAGAGCTTGGACGTGTAG	
mKctd19-m2-sgRNA3	AGTTTTTGCATGGGGGTGAC	
**Primers used for human Sanger sequencing**		
**Primer name**	**Sequence (5'- 3')**	**Product (bp)**
hKCTD19-MT1-FW	TACAGGTGTGAACCACCATG	583
hKCTD19-MT1-RV	AAGAGGGTCATCTCAGCAAC	
hKCTD19-MT2-FW	CATCCAGGTGTCCCTATGCC	497
hKCTD19-MT2-RV	TCCTCTGCTTGCTCCAGTCC	
hKCTD19-MT3-FW	GTGTCCCTATGCCGAAATGC	496
hKCTD19-MT3-RV	CCTTGGTCCTCTGCTTGCTC	
**Primers used for blood nested RT-PCR**		
**Primer name**	**Sequence (5'- 3')**	**Product (bp)**
hKCTD19-RT-MT1-1-FW	ATGGAGGAGTCTGGCATGGC	525
hKCTD19-RT-MT1-1-RV	GCAGTAGTGCACCTCCTC	
hKCTD19-RT-MT1-2-FW	CGTAGGGGGCTGGCATTTC	245
hKCTD19-RT-MT1-2-RV	GCAGCAAAGGCATCAGCTGC	
hKCTD19-RT-MT2-1-FW	CTGAGCAGTACACTCGGC	588
hKCTD19-RT-MT2-1-RV	TAACCCCTAGGCTGTCCAC	
hKCTD19-RT-MT2-2-FW	GAGAGCCCCCCAAAGAAGA	256

hKCTD19-RT-MT2-2-RV	TGGAATGGGCTTTCCAGGC	
hACTB-RT-FW	AATGAGCTGCGTGTGGCTC	148
hACTB-RT-RV	ATAGCACAGCCTGGATAGCAAC	

Primers used for plasmid construction

Primer name	Sequence (5'- 3')	Product (bp)
EGFP-BB-FW	AGCGGCCGCGACTCTAGATC	1322
EGFP-AS-RV	CAGCCGATTGTCTGTTGTGC	
EGFP-AS-FW	GCACAACAGACAATCGGCTG	3428
EGFP-BB-RV	CTTGTACAGCTCGTCCATGC	
EGFP-hKCTD19-FL-FW	GCATGGACGAGCTGTACAAGGAGGAGTCTGGCATGGC	2818
EGFP-hKCTD19-FL-RV	GATCTAGAGTCGCGGCCGCTCTAGTCCTCTTGTAGGTACT	
EGFP-hKCTD19-MT1-FW	AGGCACGTGCACACTATTACCTCTAC	3906
EGFP-hKCTD19-MT1-AS-RV	TCTGTTGTGCCCAGTCATAG	
EGFP-hKCTD19-MT1-AS-FW	CTATGACTGGGCACAACAGA	3650
EGFP-hKCTD19-MT1-RV	GTAGAGGTAATAGTGTGCACGTGCCT	
EGFP-hKCTD19-MT2-FW	GCCACTCCCATGTAAAAACTCATCT	3668
EGFP-hKCTD19-MT2/3-AS-RV	TGGTATCTGCGCTCTGCTGA	
EGFP-hKCTD19-MT2/3-AS-FW	TCAGCAGAGCGCAGATACCA	3885
EGFP-hKCTD19-MT2-RV	AGATGAGTTTTTACATGGGAGTGGC	
EGFP-hKCTD19-MT3-FW	TTGGAGGAGGCCACCCTGTAGCTCCCCTT	3566
EGFP-hKCTD19-MT2/3-AS-RV	TGGTATCTGCGCTCTGCTGA	
EGFP-hKCTD19-MT2/3-AS-FW	TCAGCAGAGCGCAGATACCA	3991
EGFP-hKCTD19-MT3-RV	AAGGGGAGCTACAGGGTGGCCTCCTCCAA	

Primers used for genotyping, RT-PCR and qPCR of *Kctd19* mutant mice

Primer name	Sequence (5'- 3')	Product (bp)

mKctd19-m1-Check-FW	GCTGGCATTTCTCAGTTCCC	247
mKctd19-m1-Check-RV	CTTCTTACCTGAAGCAAGGGC	
mKctd19-m2-Check-FW	AGAGTCCCCCCAAGAAGAAG	381
mKctd19-m2-Check-RV	TGCCCCAGTCCTTTAGGGTA	
mKctd19-m1-RT-FW	AGGACTATGGAGGAGCCTGG	341
mKctd19-m1-RT-RV	AGGTGGTGTCTCCCTTCCTT	
mKctd19-m2-RT-FW	AGGAAGCTGCATGTGGTGAC	440
mKctd19-m2-RT-RV	AGTGGCTGGAATTCCCACTG	
mActb-RT-FW	ACCAACTGGGACGACATGGAGAA	213
mActb-RT-RV	TACGACCAGAGGCATACAGGGAC	
mKctd19-m1/m2-q-FW	AGGACTATGGAGGAGCCTGG	341
mKctd19-m1/m2-q-RV	AGGTGGTGTCTCCCTTCCTT	
mActb-q-FW	ACCAACTGGGACGACATGGAGAA	213
mActb-q-RV	TACGACCAGAGGCATACAGGGAC	

Table S4. Details of filtered variants from whole-exome sequencing analysis pipeline

Case ID	Gene	Transcript ID	Mutation type	cDNA change	Remark
P7864	*LIPT2*	ENST00000310109	Frameshift	c.123_124A	Deleterious mutations in *LIPT2* have been reported to be associated with neonatal encephalopathy (PMID: 28757203).
P7864	*PARVA*	ENST00000538608	Missense	c.670G>A	Knockout *Parva* in mice causes embryonic lethality due to severe cardiovascular defects (PMID: 19798050).
P7864	*SMPD1*	ENST00000299397	Missense	c.995C>G	Deleterious mutations in *SMPD1* have been reported to be associated with Niemann-Pick disease (PMID: 2023926).
P2034	*CMC2*	ENST00000561614	Splicing	c.305+1G>A	The transcript affected by the mutation is a "nonsense mediated decay" transcript, and the mutation does not affect the classical transcript (ENST00000219400), so the gene still encodes a normal protein in P2034.
P2034	*ZNF891*	ENST00000537226	Nonframeshift	c.836_839T	According to the expression pattern of *ZNF891* in human testis single cell transcriptome data provided by FertilityOnline database, *ZNF891* is mainly expressed in spermatids (PMID: 34954426) (data is available upon request), while there are no post-meiotic cells in the testis of P2034, so it is speculated that mutation in *ZNF891* is not the cause of meiotic arrest in the patient.
P2034	*PDF*	ENST00000288022	Missense	c.8G>A	The mutated amino acid site is not evolutionarily conserved, and there is no homologous site in *Rhesus* (data is available upon request)
P2034	*BMP2K*	ENST00000502871	Missense	c.1221T>A	According to MGI database, *Bmp2k* knockout mice exhibit abnormalities in homeostasis, renal/urinary and vision/eye (MGI Direct Data Submission, PMID: 30407599).

Family-01	*FHOD1*	ENST00000258201	Splicing	c.308+3C>T	According to MGI database, *Fhod1* knockout mice exhibit abnormalities in cardiovascular and growth/size/body (MGI Direct Data Submission, PMID: 30407599).
Family-01	*DPEP3*	ENST00000268793	Missense	c. 572G>A	Male *Dpep3* knockout mice are fertile (PMID: 31212048).
Family-01	*UTP4*	ENST00000314423	Missense	c.1183T>C	According to MGI database, *Utp4* knockout mice exhibit preweaning lethality with complete penetrance (MGI Direct Data Submission, PMID: 30407599).
Family-01	*FUK*	ENST00000288078	Missense	c.766G>A	Deleterious mutations in *FUK* have been reported to be associated with congenital disorder of glycosylation (PMID: 30503518).
